# Degradation of Bile Acids by Soil and Water Bacteria

**DOI:** 10.3390/microorganisms9081759

**Published:** 2021-08-17

**Authors:** Franziska Maria Feller, Johannes Holert, Onur Yücel, Bodo Philipp

**Affiliations:** 1 Institute for Molecular Microbiology and Biotechnology, University of Münster, Corrensstr. 3, 48149 Münster, Germany; franziska.feller@uni-muenster.de (F.M.F.); johannes.holert@uni-muenster.de (J.H.); onuryuecel@googlemail.com (O.Y.); 2 Fraunhofer Institute for Molecular Biology and Applied Ecology IME, Auf dem Aberg 1, 57392 Schmallenberg, Germany

**Keywords:** steroid degradation, bile acids, bacterial metabolism, cholic, deoxycholic, chenodeoxycholic

## Abstract

Bile acids are surface-active steroid compounds with a C_5_ carboxylic side chain at the steroid nucleus. They are produced by vertebrates, mainly functioning as emulsifiers for lipophilic nutrients, as signaling compounds, and as an antimicrobial barrier in the duodenum. Upon excretion into soil and water, bile acids serve as carbon- and energy-rich growth substrates for diverse heterotrophic bacteria. Metabolic pathways for the degradation of bile acids are predominantly studied in individual strains of the genera *Pseudomonas*, *Comamonas*, *Sphingobium*, *Azoarcus*, and *Rhodococcus*. Bile acid degradation is initiated by oxidative reactions of the steroid skeleton at ring A and degradation of the carboxylic side chain before the steroid nucleus is broken down into central metabolic intermediates for biomass and energy production. This review summarizes the current biochemical and genetic knowledge on aerobic and anaerobic degradation of bile acids by soil and water bacteria. In addition, ecological and applied aspects are addressed, including resistance mechanisms against the toxic effects of bile acids.

## 1. Bile Acids and Their Occurrence in the Environment

Bile acids, or bile salts in their de-protonated state, are surface-active steroid compounds that serve many functions in the digestive tract of vertebrates [1,2] and as regulatory signaling compounds [3]. These functions and the interplay of bile acids with gastrointestinal bacteria [4,5,6,7,8,9,10] are described in the accompanying review articles of this special issue. The focus of this review is on the current knowledge about the metabolic pathways of soil and water bacteria, which use bile acids as a carbon and energy source under aerobic and anaerobic conditions and their ecological and biotechnological implications.

More than 90% of vertebrae bile acids are typically re-adsorbed from the intestine during the enterohepatic cycle. The rest is released into the environment by feces and urine. For example, humans release 400–600 mg of bile acids per person and day [4], adding up to about 180 g per year. A large input of bile acids into the environment originates from farm animals. 1 g of cow feces contains about 1 mg cholic acid and 3 mg deoxycholic acid, and 1 g chicken feces can contain up to 7.5 mg chenodeoxycholic acid [11]. Theoretically, this could lead to bile acid concentrations in the millimolar range of up to 20 mM when animal excretions are used on agricultural land as fertilizer [12]. In accordance with that, about 0.3 mM deoxycholic and about 0.5 mM chenodeoxycholic acid were detected in cow and poultry manure-runoff simulations [13], and even in rivers up to 13 nM of lithocholic acid and up to 76 nM of deoxycholic acid were detectable [14]. Bile acids are also released by aquatic vertebrates, functioning as signaling compounds [15,16].

**Figure 1:**
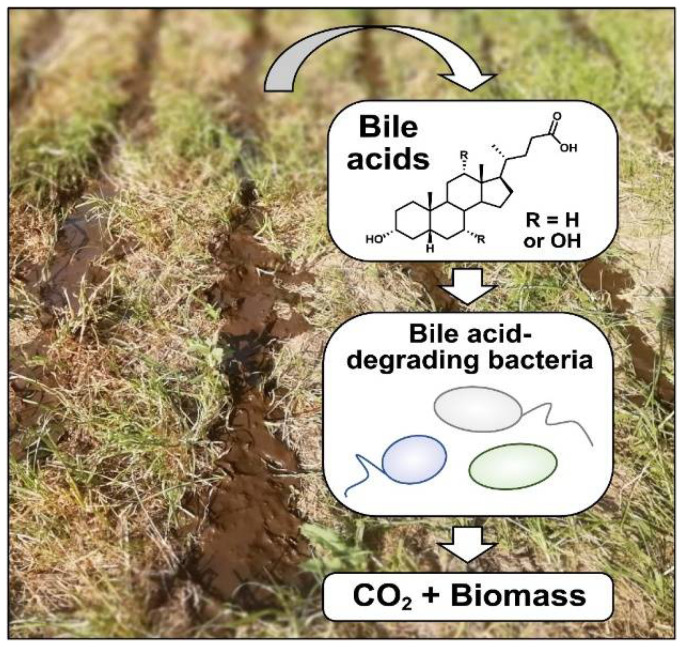
Manure fertilizer on agricultural fields as a source for bile salts, which can be used as a carbon and energy source by heterotrophic bacteria.

As a result of their widespread release by vertebrates, bile acids constitute a rich nutrient source for many bacteria in soils and water, in particular near fecal pellets of vertebrates and in manure-fertilized areas (Figure 1). In accordance with this, rapid chenodeoxycholic acid degradation was observed in soil slurries from agricultural fields that were spiked with this bile acid [12]. Bacterial growth in enrichment cultures containing bile acids as substrates inoculated with soil or freshwater usually occurs within 24–48 h and bile acid-degrading bacteria can readily be isolated from terrestrial and limnic habitats [17,18,19]. Further supporting this, a recent study analyzing steroid degradation genes in metagenomes from diverse environments revealed that soils and rhizosphere, as well as marine and selected freshwater environments, are prominent habitats for steroid-degrading bacteria [20]. As bile acids are highly reduced and carbon-rich compounds, they provide an attractive carbon- and energy source, particularly in nutrient-poor soils and waters. *Pseudomonas stutzeri* Chol1 has a molar growth yield of about 250 g dry mass per mol cholic acid under aerobic conditions [19]. This suggests that about 25 mol adenosine triphosphate (ATP) can be derived from the degradation of 1 mol cholic acid, considering that 1 mol of ATP is required for the formation of 10 g of dry mass [21].

In spite of their good degradability, bile acids can be used as paleontological markers [22] in some environments where their degradation is hindered by adverse environmental factors such as unfavorable pH, temperature, or salinity, desiccation, or a lack of suitable electron acceptors.

## 2. Bacterial Bile Acid Degradation

### 2.1. Diversity of Bile Acid-Degrading Bacteria

In recent years, many bile acid-degrading bacteria have been isolated from different environments such as meadows, freshwater, and marine habitats. All these strains belong to the Proteobacteria or Actinobacteria. Among the Proteobacteria, individual strains of the genera *Sphingomonas*, *Sphingobium*, *Novosphingobium* (*α*-Proteobacteria; ([18,23])), *Comamonas*, *Azoarcus*, *Zoogloea* (*β*-Proteobacteria; [18,24,25]), *Pseudomonas*, *Pseudoalteromonas* and *Shewanella* (*γ*-Proteobacteria; [17,19,26,27]) have been identified to be able to degrade bile acids. Among the Actinobacteria, several members of the genus *Rhodococcus* [28,29], as well as individual strains from the genera *Thermomonospora*, *Amycolatopsis* [27], *Dietzia* [18], *Gordonia* [17], and *Nocardia* [30] are able to grow with and metabolize bile acids.

The apparent restriction of bile acid degradation to the Actinobacteria and *α*-, *β*-, and *γ*-Proteobacteria is strongly supported by two recent studies analyzing the occurrence of steroid degradation genes in sequenced genomes and metagenomes [20,27], which found aerobic steroid degradation genes only in members of these taxa. Homologs of known bile acid degradation proteins were only found in genomes of the Actinobacteria genera *Rhodococcus*, *Gordonia*, and *Saccharomonospora* and in genomes of the Proteobacteria genera *Comamonas*, *Glaciecola*, *Marinobacterium*, *Pseudoalteromonas*, *Pseudomonas*, *Shewanella*, and *Sphingomonas* [27].

### 2.2. General Aspects of Metabolic Bile Acid Degradation Pathways

**Figure 2:**
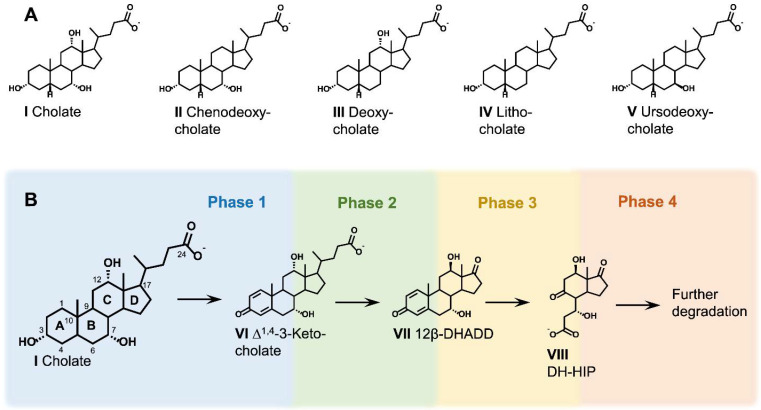
General aspects of bile acid degradation. (**A**) Structures of the most common bile acids in humans. (**B**) General scheme of bile acid degradation by bacteria. For bile acids, the respective deprotonated bile salts are depicted. Abbreviations: 12*β*-DHADD, 7*α*,12*β*-Dihydroxy-androsta-1,4-diene-3,17-dione; DH-DHIP, 3′,7-Dihydroxy-H-methyl-hexahydro-indanone-propanoate.

Bacterial bile acid degradation has primarily been studied with cholic acid (compound I) as a model substrate [31] and to a lesser extent with the differently hydroxylated bile acids chenodeoxycholic (II) and deoxycholic acid (III, Figure 2A). Degradation of lithocholic acid (IV) represents a special scenario since its A-ring oxidized derivatives are also natural intermediates of the bacterial cholesterol and phytosterol degradation pathway [32]. Transcriptomic studies in a bile acid and sterol degrading Actinobacterium suggested that lithocholic acid is degraded by a mix of cholesterol and bile acid degradation-specific reactions [30].

Aerobic degradation of cholic acid and other bile acids has predominantly been studied in *Pseudomonas stutzeri* Chol1 [31,33,34], *Pseudomonas putida* DOC21 [17,35], *Comamonas testosteroni* [24,36,37,38,39], *Sphingobium* sp. strain Chol11 [18,23,40,41,42], and *Rhodococcus jostii* RHA1 [28,43]. Complete anaerobic degradation of bile acids has been studied in the facultative anaerobic, denitrifying *β*-proteobacterium *Azoarcus* sp. strain Aa7 (Yücel et al., 2017). Genetic, biochemical, and physiological studies have shown that many bile acid degradation reactions, especially for the degradation of the steroid nucleus, are homologous to the respective reaction steps known from bacterial cholesterol or testosterone degradation pathways [24,32,44,45].

**Figure 3:**
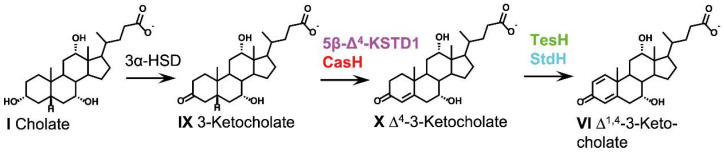
A-ring oxidation as phase 1 of bacterial bile acid degradation. For bile acids, the respective deprotonated bile salts are depicted. Text colors indicate the organism, in which the respective enzyme has been identified: light blue, *P. putida* DOC21; green, *C. testosteroni* TA441; red, *R. jostii* RHA1; magenta, *Sphingobium* sp. strain Chol11.

**Figure 4:**
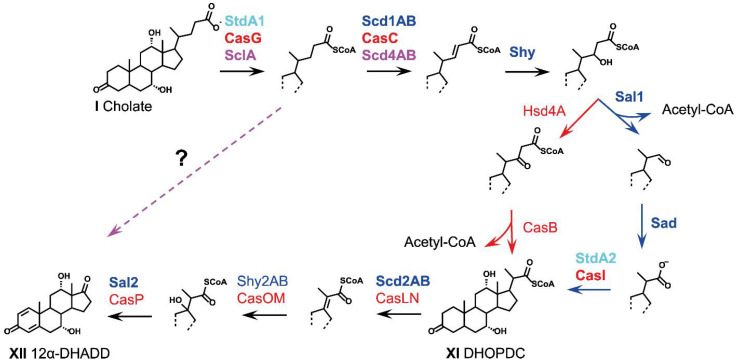
Degradation of the carboxylic C_5_ side chain as phase 2 of bacterial bile acid degradation. For bile acids, the respective deprotonated bile salts are depicted. Text and arrow colors indicate the organism, in which the respective enzyme has been identified: dark blue, *P. stutzeri* Chol1; light blue, *P. putida* DOC21; green, *C. testosteroni* TA441; red, *R. jostii* RHA1; magenta, *Sphingobium* sp. strain Chol11; black arrows, found in several organisms; dotted magenta arrow, so far unknown side-chain degradation pathway in *Sphingobium* sp. strain Chol11 and other *Sphingomonadaceae*. Abbreviations: DHOPDC, 7,12-Dihydroxy-3-oxo-pregna-1,4-diene-carboxylic acid; 12*α*-DHADD, 7*α*,12*α*-Dihydroxy-androsta-1,4-diene-3,17-dione.

**Figure 5:**
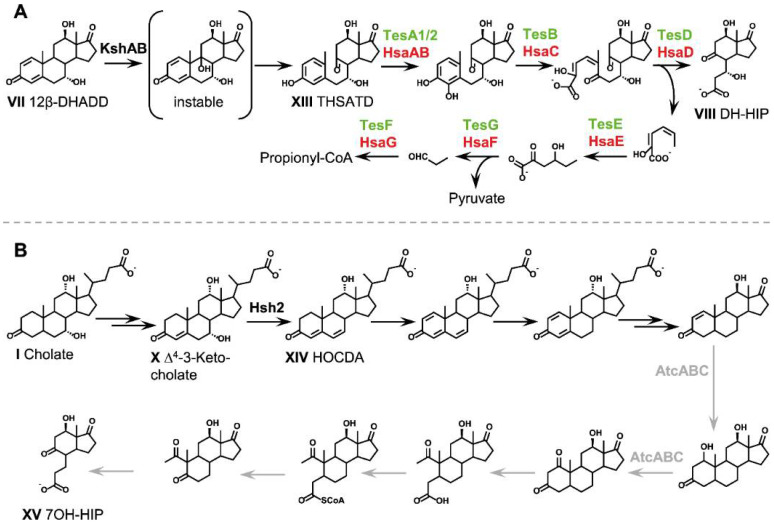
Cleavage of rings A and B as well as degradation of the A-ring as phase 3 of bacterial bile acid degradation. (**A**) Aerobic degradation via the 9,10-*seco* pathway. Text colors indicate the organism, in which the respective enzyme has been identified: dark blue, *P. stutzeri* Chol1; light blue, *P. putida* DOC21; green, *C. testosteroni* TA441; red, *R. jostii* RHA1; magenta, *Sphingobium* sp. strain Chol11; black, identified in several organisms. (**B**) Anaerobic degradation via the 2,3-*seco* pathway. Colors: black, described for *Azoarocus* sp. strain Aa7; grey, as inferred from anaerobic degradation of cholesterol and testosterone. For bile acids, the respective deprotonated bile salts are depicted. Abbreviations: HOCDA, 12*α*-Hydroxy-3-oxo-4,6-choldienoic acid; 7OH-HIP, 7-Hydroxy-H-methyl-hexahydro-indanone-propanoate.

**Figure 6:**
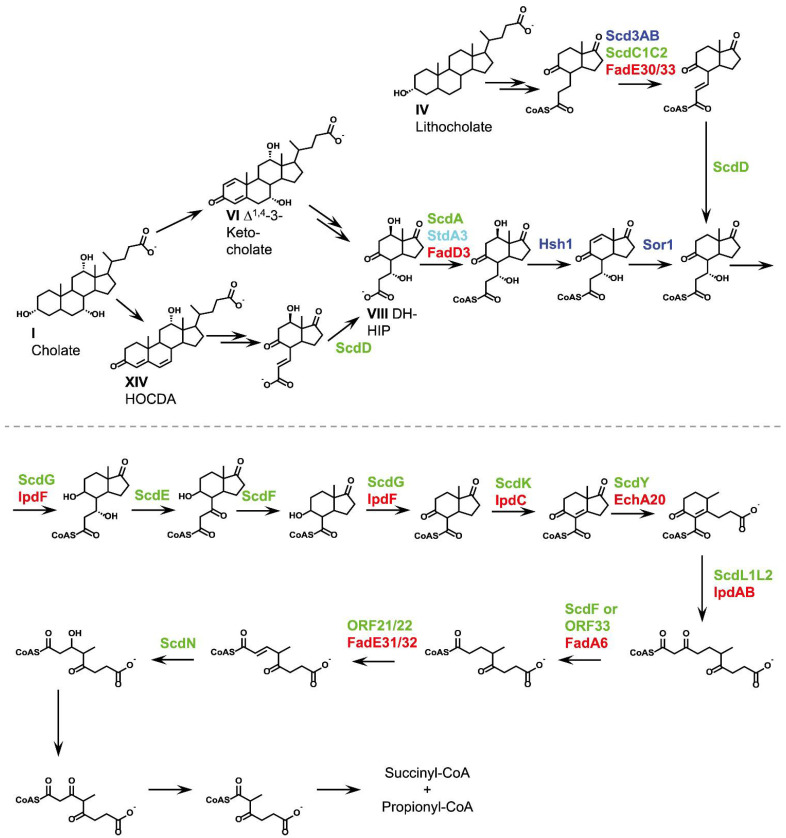
Degradation of H-methyl-hexahydro-indanone-propanoate (HIP) derivatives as phase 4 of bacterial bile acid degradation and channeling of differently hydroxylated bile acids into one common pathway via Δ^1,4^-3-keto intermediates or Δ^4,6^-intermediates. For bile acids, the respective deprotonated bile salts are depicted. Text colors indicate the organism, in which the respective enzyme has been identified: dark blue, *P. stutzeri* Chol1; light blue, *P. putida* DOC21; green, *C. testosteroni* TA441; red, *R. jostii* RHA1; magenta, *Sphingobium* sp. strain Chol11.

Bile acid degradation can be divided into four phases (Figure 2B): (1) partial oxidation of the A-ring (Figure 3), (2) stepwise removal of the C_5_ carboxylic side chain, including the release of an acetyl-CoA and a propionyl-CoA residue (Figure 4), (3) successive opening of the B- and A-ring and degradation of the former A-ring (Figure 5), and (4) degradation of the remaining C/D-rings (Figure 6). While this unifying scheme appears to occur in most bile acid-degrading bacteria, some individual degradation pathways exhibit specific differences in the order of the reaction sequences [43] and in individual biochemical reactions [40]. The A-ring oxidation, side chain, and C/D-ring degradation phases do not require elemental oxygen as co-substrate and are largely homologous in the anaerobic and aerobic degradation pathways. However, cleavage of the B- and A-ring (phase 3) differs significantly between aerobic and anaerobic conditions: elemental oxygen is used by oxygenases to open the steroid ring system under aerobic conditions [46,47], while molybdoenzymes catalyze a water-based ring-opening under anaerobic conditions. The respective aerobic and anaerobic degradation pathways of the steroid nucleus are known as the 9,10-*seco* pathway [48] and the 2,3-*seco* pathway [25,49,50], respectively. The facultatively denitrifying *Azoarcus* sp. strain Aa7 is the first reported bile acid-degrading bacterium that uses either the 9,10-*seco* or the 2,3-*seco* pathway, depending on the availability of oxygen [25].

In general, bile acid degradation genes are organized in large gene clusters [24,27,28,32,51], which often contain sub-clusters for individual reaction steps such as side chain and ring degradation (Table 1). The genomes of many bile acid-degrading Proteobacteria have a single gene cluster for bile acid degradation as found in *P. stutzeri* Chol1 [51] and *C. testosteroni* TA441 [39,52]. Others have multiple distinct gene clusters, such as *Pseudoalteromonas haloplanktis* [27] or *Azoarcus* sp. strain Aa7 (Yücel et al., 2017). In *Sphingobium* sp. strain Chol11, three clusters and several single genes have been identified to be involved in bile acid metabolism [23,41]. The genome of *R. jostii* RHA1 comprises two distinct gene clusters for cholic acid and cholesterol degradation, which are differentially expressed depending on the steroid carbon source [28]. However, genes involved in the C/D-ring degradation of cholic acid and cholesterol are only found in the cholesterol degradation cluster.

Genes and proteins characterized and predicted to be involved in bile acid degradation in P. stutzeri Chol1, C. testosteroni CNB-2, Sphingobium sp. strain Chol11, and R. jostii RHA1 are summarized in Table 1.

**Table 1:** Characterized and predicted bile acid degradation genes (locus tags) located in the steroid degradation gene clusters of *Pseudomonas stutzeri* Chol1, *Comamonas testosteroni* CNB-2, *Rhodococcus jostii* RHA1, and *Sphingobium* sp. strain Chol11. Protein names are given in parentheses when available and are taken from the corresponding literature. Characterized genes and proteins are bold.

Bile Acid Degradation Pathway	Function	*P. stutzeri* Chol1	*C. testosteroni* CNB-2 ***	*R. jostii* RHA1	*Sphingobium* sp. Strain Chol11
**Cleavage of conjugated bile acids**	Bile salt amidase	C211_RS11020	CTCNB1_RS06560 (ORF26) CTCNB1_TS06555 (ORF25)	unknown (RHA1_RS22310)	**Nov2c227 (Bsa)**
**C5 side-chain degradation**	CoA-ligase	C211_RS11125 (StdA1 *)	CTCNB1_RS06840	**RHA1_RS28415 (CasG)**	**Nov2c230 (SclA)**
	ACADs	**C211_RS11115 (Scd1A)**	CTCNB1_RS06830	**RHA1_RS28395 (CasC)**	**Nov2c221 (Scd4A)**
		**C211_RS11120 (Scd1B)**	CTCNB1_RS06835	---	**Nov2c222 (Scd4B)**
	Enoyl-CoA hydratase	**C211_RS11210 (Shy1)**	CTCNB1_RS06880	RHA1_RS28400 (CasD)	absent
	Steroid aldolase	**C211_RS11205 (Sal1)**	CTCNB1_RS06875	absent	absent
	Steroid aldehyde dehydrogenase	**C211_RS11010 (Sad)**	CTCNB1_RS06680	absent	absent
	2-hydroxy-CoA dehydrogenase	absent	absent	RHA1_RS28410	absent
	Steroid thiolase	absent	absent	RHA1_RS28390 (CasB)	absent
**C3 side-chain degradation**	CoA-ligase	C211_RS11185 (StdA2 *)	CTCNB1_RS06820	**RHA1_RS28425 (CasI)**	absent
	ACADs	C211_RS11105 (Scd2A)	CTCNB1_RS06815	RHA1_RS28440 (CasL)	absent
		**C211_RS11090 (Scd2B)**	CTCNB1_RS06800	RHA1_RS28450 (CasN)	absent
	Enoyl-CoA hydratase	**C211_RS11085 (Shy2)**	CTCNB1_RS06795	RHA1_RS28445 (CasM)	absent
		C211_RS11095	CTCNB1_RS06805	RHA1_RS28455 (CasO)	absent
	Steroid aldolase	C211_RS11100 (Sal2)	CTCNB1_RS06810	RHA1_RS28460 (CasP)	absent
**A-ring oxidation**	3*α*-Hydroxysteroid dehydrogenase	C211_RS10975	**CTCNB1_RS06750** (3*α*-HSD)	unknown	Nov2c6
	∆^1^-Ketosteroid dehydrogenase	**C211_RS11030 (** **Δ^1^-KstD)**	**CTCNB1_RS06925 (TesH)**	RHA1_RS28305 (KstD3)	Nov2c82
		---	---	RHA1_RS28380 (KstD3b)	---
	Δ^4^-5*α*-Ketosteroid dehydrogenase	C211_RS11110	**CTCNB1_RS06930 (TesI)**	RHA1_RS27810	Nov2c17
	Δ^4^-5*β*-Ketosteroid dehydrogenase	Unknown	CTCNB1_RS06740	**RHA1_RS28420 (CasH)**	**Nov2c19** **(** **Δ^4^-5*β*-KSTD1)**
**9,10-*seco* pathway**	3-Ketosteroid-9*α* hydroxylase, oxygenase	**C211_RS11300**	CTCNB1_RS06665	**RHA1_RS28370 (KshA3)**	**Nov2c407**
		---	---	---	**Nov2c430**
		---	---	---	**Nov2c440**
	3-Ketosteroid-9*α* hydroxylase, reductase	**C211_RS11040**	CTCNB1_RS06935 (ORF17)	RHA1_RS28480 (KshB3)	absent
	9,10-*seco*-steroid hydroxylase, oxygenase	C211_RS11025	**CTCNB1_RS06920 (TesA2)**	RHA1_RS28325 (HsaA3)	Nov2c349
	9,10-*seco*-steroid hydroxylase, reductase	C211_RS11005	**CTCNB1_RS06915 (TesA1)**	RHA1_RS28295 (HsaB3)	absent (Nov2c347)
	9,10-*seco*-steroid dioxygenase	C211_RS11215	**CTCNB1_RS06510 (TesB)**	RHA1_RS28330 (HsaC3)	Nov2c350
	4,5,9,10-di*seco*-steroid hydroxylase	C211_RS11155	**CTCNB1_RS06910 (TesD)**	RHA1_RS28300 (HsaD3)	Nov2c348
**C/D-ring (HIP) side-chain degradation**	CoA-ligase	C211_RS11045 (StdA3 *)	**CTCNB1_RS06940 (ScdA)**	RHA1_RS22410 ** (FadD3)	Nov2c359
	ACAD	**C211_RS11065 (Scd3A)**	**CTCNB1_RS06570 (ScdC1)**	RHA1_RS22390 **	Nov2c367
		**C211_RS11070 (Scd3B)**	**CTCNB1_RS06575 (ScdC2)**	RHA1_RS22415 **	Nov2c361
	Enoyl-CoA hydratase	C211_RS11075	**CTCNB1_RS06585 (ScdD)**	unknown	Nov2c364
		C211_RS11270	---	---	---
	2-hydroxy-acyl-CoA dehydrogenase	C211_RS11260	**CTCNB1_RS06565 (ScdE)**	RHA1_RS22710 **	Absent (Nov2c360, Nov2c362)
	HIP thiolase	C211_RS11275	CTCNB1_RS06590 (ORF33)	RHA1_RS28520	Nov2c358
**C/D-ring (HIC) degradation**	5-OH HIC-CoA reductase	C211_RS11235	**CTCNB1_RS06530 (ScdK)**	RHA1_RS22685 ** (IpdC)	Nov2c354
	5-Oxo HIC-CoA oxidase	C211_RS11265	**CTCNB1_RS06580 (ScdG)**	RHA1_RS22420 ** (IpdF)	Nov2c363
	HIEC-CoA hydrolase	unknown	**CTCNB1_RS06535 (ScdY)**	RHA1_RS27700 ** (EchA20)	Nov2c355
	COCHEA-CoA hydrolase, *α*-subunit	C211_RS11220	**CTCNB1_RS06515 (ScdL1)**	RHA1_RS22695 ** (IpdA)	Nov2c351
	COCHEA-CoA hydrolase, *β*-subunit	C211_RS11225	**CTCNB1_RS06520 (ScdL2)**	RHA1_RS22690 ** (IpdB)	Nov2c352
	*β*-Keto CoA thiolase	C211_RS11255	**CTCNB1_RS06550 (ScdF)**	RHA1_RS22430 ** (FadA6)	Nov2c366
	ACAD	C211_RS11245	CTCNB1_RS06540 (ORF21)	RHA1_RS22400 ** (FadE31)	Nov2c356
	ACAD	C211_RS11250	CTCNB1_RS06545 (ORF22)	RHA1_RS22395 ** (FadE32)	Nov2c357
	Hydratase	C211_RS11230	**CTCNB1_RS06525 (ScdN)**	RHA1_RS22405	Nov2c353
**2-hydroxy-hexa-2,4-dienoate degradation**	2-Hydroxypenta-2,4-dienoate hydratase	C211_RS10995	**CTCNB1_RS06905 (TesE)**	RHA1_RS28310 (HsaE3)	Nov2c346
	Acetaldehyde dehydrogenase	C211_RS10985	**CTCNB1_RS06895 (TesG)**	RHA1_RS28315 (HsaG3)	Nov2c344
	4-Hydroxy-2-ketovalerate aldolase	C211_RS10990	**CTCNB1_RS06900 (TesF)**	RHA1_RS28320 (HsaF3)	Nov2c345
**Transformation of 12OH**	12*α*-Hydroxy-steroid dehydrogenase	C211_RS11165	**CTCNB1_RS06490 (SteA)**	unknown	Nov2c15
	12-Keto-steroid hydrogenase	C211_RS11170	**CTCNB1_RS06495 (SteB)**	unknown	Nov2c16
	12*β*-hydroxy steroid dehydratase	**C211_RS11175 (Hsh1)**	CTCNB1_RS06500 (ORF7)	unknown	Nov2c12
	Steroid oxidoreducatse	**C211_RS11180 (Sor1)**	CTCNB1_RS06505 (ORF6)	unknown	Nov2c13
**Δ^4,6^ variant**	7*α*-Hydroxysteroid dehydratase	absent	absent	absent	**Nov2c400 (Hsh2)**

* Protein names from *P. putida* DOC21; ** genes located in cholesterol-degradation gene cluster of RHA1; *** characterized steroid degradation genes in *C. testosteroni* TA441 have been mapped to strain CNB-2 [24].

### 2.3. Uptake of Bile Acids

Bile acid substrates have to be transported into the cytoplasm before their complete mineralization. The knowledge about the transport of bile acids in bacteria is very limited. Below their critical micellar concentration, bile acids can passively enter the bacterial cell via trans-bilayer movement [53]. In their protonated, uncharged state, bile acids can undergo transverse diffusion across lipid membranes, in which the diffusion rate is determined by the number and position of the hydroxy groups on the steroid skeleton. In addition, bile acids can cross lipid membranes via transmembrane proteins. Deletion of *ompF* encoding a porin protein in an *Escherichia coli* strain resulted in a less susceptible phenotype towards the toxic effects of bile acids, suggesting that bile acids can pass across the membrane via protein channels like OmpF [54]. The membrane transport protein BaiG from the intestinal bile acid-dehydroxylating bacterium *Clostridium scindens* VPI12708 has proton-motive-force-dependent bile acid uptake activity [55]. Heterologous expression of BaiG in *E. coli* causes increased uptake of cholic acid, chenodeoxycholic acid, and deoxycholic acid.

However, bile acid uptake and transport systems in bile acid-degrading bacteria are still largely unknown. In *Sphingobium* sp. strain Chol11, several TonB-dependent receptor proteins were more abundant in bile acid grown cells compared to glucose grown cells, suggesting that TonB-dependent receptors could be involved in bile acid uptake in this strain [23]. In support of this, a TonB-dependent receptor was recently shown to be involved in bacterial steroid transport in the *α*-Proteobacterium *Caenibius tardaugens* NBRC16725 (formerly *Novosphingobium tardaugens*), in which a TonB deletion mutant showed reduced growth with estradiol [56]. Furthermore, it has been shown that expression of the porin-encoding gene *rjpA* is upregulated in *R. jostii* RHA1 grown with cholic acid and that RjpA contributes to cholic acid uptake in this strain [57]. In addition, an ABC transporter encoded by *camABCD* and an MFS transporter encoded by *camM* in strain RHA1 function in the uptake of cholic acid metabolites with an aromatized A-ring or with perhydroindane structures, respectively [43]. The ABC transporter system Mce4, which is involved in the trans-membrane transport of cholesterol and other hydrophobic sterols in steroid-degrading Actinobacteria, is not involved in the uptake of cholic acid [58,59].

### 2.4. Oxidative Reactions at the A-Ring (Phase 1)

Partial oxidation of the A-ring including oxidation of the 3-hydroxy group to a keto-group and desaturation of the ring between C1 and C2 (Δ^1^) and between C4 and C5 (Δ^4^) are the first enzymatic steps during both aerobic and anaerobic degradation of bile acids (Figure 3) [24,25,28,31,42].

#### 2.4.1. 3*α*-Hydroxysteroid Dehydrogenases

The degradation of bile acids is initiated by 3*α*-hydroxysteroid dehydrogenases (3*α*-HSD), yielding 3-keto-bile acids. For the degradation of cholic acid (compound I), this reaction leads to 3-ketocholic acid (IX). 3*α*-HSDs belong to the short-chain dehydrogenase protein family (SDR) or aldo-keto reductase superfamily. Bacterial 3*α*-HSDs are best studied in steroid-degrading *C. testosteroni* strains and have been shown to catalyze a bidirectional NAD(P)^+^/NADP(H)-dependent oxidoreduction of 3*α*-hydroxy- or 3-keto-groups of a variety of steroid substrates and of several other xenobiotic carbonyl compounds [60,61]. For *P. stutzeri* Chol1 and *Sphingobium* sp. strain Chol11, NAD^+^ dependent 3*α*-HSD activity has been detected in cell extracts with cholic acid [26,40]. The formation of 3-keto intermediates in other bile acid-degrading bacteria suggests that this step is conserved in aerobic and anaerobic bile acids degradation [25,62]. 3*α*-HSDs are also found in bacteria, which cannot utilize steroids as growth substrates, e.g., *P. aeruginosa* [62,63].

#### 2.4.2. 3-Ketosteroid Dehydrogenases

Further oxidation of the A-ring is catalyzed by 3-ketosteroid dehydrogenases, which catalyze Δ^1^- and Δ^4^-dehydrogenation reactions, introducing double bonds into the A-ring. In the degradation of cholic acid, these reactions lead to Δ^4^-3-ketocholate (X) and Δ^1,4^-3-ketocholate (VI).

Mammalian and many other bile acids have a bent steroid nucleus with rings A and B in *cis* configuration and a 5*β* conformation [1]. The respective 5*β*-3-keto bile acids produced by 3*α*-HSDs are oxidized by 5*β*-Δ^4^-3-ketosteroid dehydrogenases (5*β*-Δ^4^-KSTDs), which introduce Δ^4^ double bonds leading to a flat steroid nucleus. One 5*β*-Δ^4^-KSTD has been purified from *C. testosteroni* and was shown to oxidize steroids without or with a C_2_ side chain [64]. Recently, 5*β*-Δ^4^-KSTD1 from *Sphingobium* sp. strain Chol11 has been shown to oxidize differently hydroxylated 5*β* bile acid derivatives with and without side chain, but no 5*α* steroids, and CasH from *R. jostii* RHA1 was shown to have the same activity [42]. These enzymes are large flavoenzymes of the Old Yellow Enzyme family that can use quinones, phenazine methosulfate, 2,6-dichlorophenolindophenol, and K_3_Fe(CN)_6_ as electron acceptors, indicating that quinones could be the natural electron acceptors [42,64]. 5*β*-Δ^4^-KSTD1 from *Sphingobium* sp. strain Chol11 contains FAD as well as FMN and an iron-sulfur cluster and is a very fast enzyme, which shows pronounced substrate inhibition [42,65]. 5*β*-Δ^4^-dehydrogenation activity has been detected in cell-free extracts of *P. stutzeri* Chol1 and *Sphingobium* sp. strain Chol11 with artificial electron acceptors K_3_Fe(CN)_6_ or phenazine methosulfate [19,40]. The formation of Δ^1/4^- and Δ^1,4^-3-keto intermediates in other organisms confirms these steps for aerobic and anaerobic bile acids degradation [18,19,24,36,40,41,43].

In contrast to 5*β* steroids, 5*α* steroids have a planar steroid nucleus. 5*α* bile acids can be found in some fish, birds, or lizards [1]. 5*α*-Δ^4^-3-ketosteroid dehydrogenases have been characterized in *C. testosteroni* (TesI) [66], *R. jostii* RHA1 (KstD) [67], and *Rhodococcus erythropolis* SQ1 (KstD4) [68] but have not been investigated in the context of bile acid degradation so far.

A second double bond is introduced into the A-ring at C1 by Δ^1^-3-ketosteroid dehydrogenases [69]. Genes encoding these enzymes are found in diverse steroid-metabolizing bacteria. A Δ^1^-3-ketosteroid dehydrogenase for bile acid degradation is encoded by the gene *stdH* in *Pseudomonas putida* DOC21 [70]. The deletion mutant *P. stutzeri* Chol1 Δ*kstD1* accumulates compounds without a Δ^1^ double bond [42]. In *C. testosteroni*, the Δ^1^-3-ketosteroid dehydrogenase TesH is encoded directly adjacent to the 5*α*-Δ^4^-3-ketosteroid dehydrogenase TesI [66,71]. Steroid-degrading Actinobacteria often encode several isoenzymes of Δ^1^-3-ketosteroid dehydrogenases [72,73,74]. Two genes (*kstD3* and *kstD3b)*, located in the cholic acid degradation gene cluster of *R. jostii* RHA1, encode putative Δ^1^-3-ketosteroid dehydrogenases [28]. The crystal structure of the 3-ketosteroid Δ^1^-dehydrogenase from *R. erythropolis* SQ1 revealed a flavin adenine dinucleotide (FAD)-binding site [75]. The dehydrogenation mechanism mediated by ∆^1^-dehydrogenases is based on the trans-axial removal of the C1(*α*) and C2(*β*) hydrogen atoms [75,76,77].

### 2.5. Side-Chain Degradation (Phase 2)

The side chains of bile acids are generally degraded in a stepwise fashion resembling in large parts *β*-oxidative reaction sequences similar to the sterol side chain degradation pathway (Figure 4). Nevertheless, some key reactions for the degradation of bile acid side chains are different from this canonical progression and vary among the different model organisms. In contrast to the degradation of the aliphatic sterol side chain, the carboxylic bile acid side chain does not need to be oxidized prior to degradation. Thus, the complete removal of the bile acid side chain is possible without elemental oxygen as a co-substrate and side-chain degradation under aerobic and anaerobic conditions seems to follow the same progression [19,25]. In contrast to bile acid degraders from the *β*- and *γ*-Proteobacteria and Actinobacteria, bile acid-degrading *α*-Proteobacteria from the *Sphingomonadacea* family do not encode the majority of side-chain degradation proteins known from other model organisms. These bacteria seem to have evolved a different progression for side-chain degradation, which is catalyzed by yet unknown enzymes [23,41,78] (preprint).

#### 2.5.1. Cleavage of Conjugated Bile Acids

Prior to excretion into the duodenum, bile acids are conjugated with the amino acids glycine or taurine via amide bonds to enhance solubility [4]. Many intestinal, non-bile acid-degrading bacteria are able to deconjugate these bile acid amides using bile salt hydrolases (Bsh) [6,79]. Additionally, several bile acid-degrading bacteria have also been reported to utilize these conjugated bile acids as growth substrates: While *C. testosteroni* strains such as KF-1 are able to utilize the complete taurocholate molecule, *Pseudomonas* and *Rhodococcus* strains were described to only degrade the bile acid moiety after cleaving the amide bond of taurocholate [80]. Similarly, *Sphingobium* sp. strain Chol11 is able to grow with both glycocholate and taurocholate, but not with the free amino acids taurine and glycine [23]. The bile acid amidase Bsa from strain Chol11 was shown to cleave both conjugated bile acids and was present in higher abundances during growth with bile acids. Interestingly, this enzyme is significantly different from the well-known bile salt hydrolase enzymes in intestinal bacteria [81].

#### 2.5.2. Coenzyme-A Activation

In analogy to the *β*-oxidation of fatty acids, coenzyme A (CoA) activation of the carboxylic group of free bile acids is the initial side-chain degradation step [26,35,41]. StdA1, a CoA-ligase encoded in the bile acid degradation gene cluster of *P. putida* DOC21 converted cholic acid and the degradation intermediates 3-ketocholic acid (IX), Δ^1/4^-3-ketocholic acid (e.g., X) and Δ^1,4^-3-ketocholic acid (VI) into the corresponding CoA-thioesters, while it did not activate bile acid derivatives with a C_3_ side chain [35]. An ortholog of StdA1, CasG, encoded in the cholic acid degradation gene cluster of *R. jostii* RHA1, showed analogous substrate preferences towards C_5_ bile acids side chains [28,82]. In cell extracts of *P. stutzeri* Chol1, both, cholic acid and Δ^1,4^-3-ketocholic acid can be activated with CoA [26] and deletion of the *stdA1* homolog in this strain inhibits side-chain degradation [42]. A homolog of StdA1 was also identified in the bile acid degradation gene cluster of *Azoarcus* sp. strain Aa7 [25], and a CoA-ligase with high specificities for the activation of C_5_ carboxylic side chains was characterized in the cholesterol degrading *β*-Proteobacterium *Sterolibacterium denitrificans* [83], suggesting that CoA-ligation is also the first step of bile acid degradation under anaerobic conditions.

While the length of the side chain appears to be the determining factor for the substrate preferences of StdA1 and CasG, the configuration of the steroid nucleus seems to be less important. In contrast, the C_5_ side chain CoA-ligase SclA from *Sphingobium* sp. strain Chol11 preferentially activates bile acid derivatives with oxidized A- and B-rings with a 3-keto-∆^4^- or 3-keto-Δ^4,6^-structure (e.g., XIV) [41] further supporting the presence of a distinct pathway among the *Sphingomonadaceae.*

#### 2.5.3. *α*,*β*-Dehydrogenation

Following CoA-activation, a double bond is introduced into the side chain by acyl-CoA-dehydrogenases (ACADs). In *P. stutzeri* Chol1, this *α,β-*dehydrogenation reaction is catalyzed by Scd1AB [84]. Scd1A and Scd1B are encoded by two adjacent genes and have been suggested to form a *α*_2_*β*_2_-heterotetrameric structure based on comparisons with other steroid ACADs. This unprecedented *αβ*-dehydrogenase architecture was initially identified in the two heteromeric ACAD complexes ChsE4/5 and ChsE1/2 in *Mycobacterium tuberculosis* H37Rv, which function in the *α,β-*dehydrogenation of C_8_ and C_3_ side-chain CoA-esters during cholesterol degradation [85,86]. In these specialized ACADs, the heterotetrameric form is essential for catalytic activity. Only one of the subunits in each *αβ-*heterodimer contains a conserved, catalytically active glutamate residue and binding of one FAD co-factor occurs at the dimer interface. Thus, each *α*_2_*β*_2_*-*heterotetramer contains two active sites and two FAD-binding sites. The *α*_2_*β*_2_-heterotetrameric structure was confirmed for a bile acid-specific ACAD in the bile acid-degrading thermophilic Actinobacterium *Thermomonospora curvata*, which showed substrate preferences for C_3_ over C_5_ side chains [87].

The steroid ACAD CasC from *R. jostii* RHA1 was shown to catalyze side chain *α,β-*dehydrogenation of the CoA-esters of cholic acid, deoxycholic acid, and 3*β*-hydroxy-5-ene-cholic acid but not of derivatives with shorter side chains [88]. CasC consists of two fused ACAD domains, of which only the C-terminal domain contains the catalytic glutamate base, while the N-terminal domain is required for FAD co-factor binding.

The genome of *Sphingobium* sp. strain Chol11 does not encode orthologs of CasC or Scd1AB based on reciprocal BLASTp analyses [41]. However, the enzyme complex Scd4AB, which has high similarities to ACADs involved in the degradation of the steroid nucleus, also catalyzes *α*,*β-*dehydrogenation of the C_5_ side chain [78] (preprint).

#### 2.5.4. Hydroxylation of the Double Bond

The *αβ-*double bond is subsequently hydrated by enoyl-CoA-hydratases, forming *β*-hydroxyacyl-CoA-thioesters. In *P. stutzeri* Chol1, the respective Δ^22^-enoyl-CoA hydratase is encoded by *shy1* [34]. Shy1 contains a C-terminal hot-dog-fold domain, which is also found in (*R*)-specific enoyl-CoA hydratases catalyzing the hydration of trans-2-enoyl-CoA to (R)-3-hydroxyacyl-CoA [89]. Homologs of Shy1 have been identified in *P. putida* DOC21 and several *C. testosteroni* strains [34,35], but are missing in *Sphingobium* sp. strain Chol11 and related bile-acid degrading *Sphingomonadaceae*. In *R. jostii* RHA1, the gene *casD* located adjacent to *casC* in the cholic acid degradation gene cluster was predicted to encode a C_5_ side chain enoyl-CoA-hydratase [28] and expression of *casD* was upregulated in the presence of cholic acid [90]. Nevertheless, the function of this gene has still to be confirmed.

#### 2.5.5. Release of Acetyl-CoA and Formation of C_3_-Carboxylic Side Chains

During fatty acid degradation, the corresponding *β*-hydroxy group is typically oxidized to a *β*-keto group. Subsequent thiolytic cleavage releases acetyl-CoA, thus producing a shortened CoA-activated fatty acid. Similarly, the release of acetyl-CoA from C_5_ *β*-hydroxy side chain degradation intermediates seems to be a general theme during bile acid degradation. Accordingly, analysis of the wild type and mutant strains of *P. stutzeri* Chol1 [26], *R. jostii* RHA1 [43], and *C. testosteroni* TA441 [36] revealed that carboxylic C_3_ side chain intermediates such as 7,12-dihydroxy-3-oxo-pregna-1,4-diene carboxylic acid (DHOPDC, XI) for cholate are formed in all these organisms. This was also shown for anaerobic and aerobic cultures of *Azoarcus* sp. strain Aa7 growing with cholic acid (Yücel et al., 2018).

In Proteobacteria, the deacetylation of the carboxyl side chain differs from the expected thiolytic cleavage known from conventional *β*-oxidation of carboxylic acids. Instead, this reaction proceeds via an aldolytic cleavage of acetyl-CoA from the C_5_ side chain yielding a free aldehyde as a product [33]. This was first identified in *P. stutzeri* Chol1, and the gene product of *sal1* was shown to catalyze the respective aldolytic cleavage reaction [34]. Sal1 shows similarities to the SCP-x-type thiolase family, but lacks a catalytically active N-terminal cysteine, which is highly conserved in catabolic thiolases [91] due to its requirement for a nucleophilic attack on the respective *β*-keto groups allowing subsequent addition of CoA. Homologs of Sal1 are also encoded in the respective bile acid degradation gene clusters of *P. putida* DOC21, *C. testosteroni*, and *Azoarcus* sp. strain Aa7, suggesting that acetyl-CoA is removed by an analogous aldolytic cleavage reaction in these strains [25,34]. No homologs of Sal1 are encoded in the genomes of *Sphingobium* sp. strain Chol11 [41] or *R. jostii* RHA1. However, the cholic acid degradation gene cluster of strain RHA1 encodes CasB [28], a homolog of the cysteine-containing thiolase FadA5, which catalyzes a thiolytic cleavage reaction of acetyl-CoA from C_5_ side-chain degradation intermediates of cholesterol in *M. tuberculosis* H37Rv [92,93]. The catalytically active cysteine of typical thiolases is also conserved in CasB, indicating that acetyl-CoA is thiolytically cleaved during bile-acid degradation in strain RHA1 [28].

In *P. stutzeri* Chol1, the C_3_ side chain aldehyde product is further oxidized to the corresponding carboxylic acid by an NAD^+^-dependent steroid aldehyde dehydrogenase encoded by the gene *sad* [34]. In the degradation of cholic acid, this reaction leads to DHOPDC (XI). Homologs of Sad are also encoded in the bile acid degradation gene clusters of *P. putida* DOC21, *C. testosteroni*, and *Azoarcus* sp. strain Aa7 [25,34], while no homologs of Sad exist in proximity to the cholic acid degradation gene cluster of *R. jostii* RHA1 or in the genome of *Sphingobium* sp. strain Chol11 and related bile-acid degrading *Sphingomonadaceae*.

An aldolytic cleavage of acetyl-CoA during bile acid degradation is unexpected because thiolysis mediated by a *β*-keto intermediate is structurally possible with C_5_ side chains. In addition, thiolysis would save energy because a subsequent ATP-dependent CoA-activation of the resulting C_3_ side chain is not necessary. Nevertheless, an aldolytic cleavage seems to be the primary reaction mechanism in bile acid-degrading *β*- and *γ*-Proteobacteria.

#### 2.5.6. Release of Propionyl-CoA and Formation of Androstadienediones (ADDs)

Further side-chain degradation follows a similar progression, leading to the release of propionyl-CoA and the formation of C_19_ steroids. In *β*- and *γ*-Proteobacteria, the first step is the CoA-activation of the C_3_ carboxylic side chain. The steroid CoA-synthetase StdA2, specific for activating carboxylic C_3_ side chain intermediates has been identified in *P. putida* DOC21 [35]. ATP-dependent CoA-activation of a cholic acid degradation intermediate with a C_3_ side chain has been measured in cell extracts of *P. stutzeri* Chol1 [26]. In *R. jostii* RHA1, the acyl-CoA-synthetase CasI encoded in the cholic acid degradation gene cluster of *R. jostii* RHA1 specifically activates steroid C_3_ side chains [28,94]. A homolog of StdA2 was also identified in the bile acid degradation gene cluster of *Azoarcus* sp. strain Aa7 and a CoA-ligase specifically activating C_3_ carboxylic side chains was characterized in *Sterolibacterium denitrificans* [25,83], suggesting that CoA-ligation of C_3_ carboxylic side chains also occurs under anaerobic conditions.

A distinct gene cluster encoding a heteromeric ACAD, a heteromeric enoyl-CoA-hydratase, and an aldolase, which together catalyze the remaining C_3_ side-chain degradation steps, seems to be conserved with similar gene synteny throughout most steroid side chain-degrading organisms [95], including *P. stutzeri* Chol1, *C. testosteroni* CNB-2, and *R. jostii* RHA1. The next step in the degradation of the C_3_ side chain of bile acids after CoA-activation is a dehydrogenation reaction catalyzed by a heteromeric ACAD belonging to the same family as the C_5_ side chain ACADs described above. The heteromeric character of these C_3_ side chain-specific ACADs was first identified in the ACAD complex ChsE1/2, which catalyzes a homologous reaction during cholesterol side-chain degradation in *M. tuberculosis* [96]. Homologs of *chsE1* and *chsE2* are also encoded in the bile acid degradation gene clusters of *P. stutzeri* Chol1, *C. testosteroni* CNB-2, and *R. jostii* RHA1. A transposon mutant of the *α*-subunit of the respective ACAD in *P. stutzeri* Chol1 (Scd2AB) was unable to degrade C_3_ side chain intermediates during cholic acid degradation [26].

The resulting double bond is subsequently hydrated, followed by the release of propionyl-CoA by a retro-aldol reaction as proposed by [97]. These reactions are catalyzed by a protein complex formed between a heteromeric enoyl-CoA-hydratase and a single aldolase in the bile acid-degrading Actinobacterium *T. curvata* [98] and during cholesterol side-chain degradation in *M. tuberculosis* [95,99,100]. The heteromeric enoyl-CoA-hydratase consists of two subunits, which both carry MaoC-like domains, and the *α*-subunit carries an additional DUF35 domain. The hydration of the C_3_ side chain double bond was shown to have an unfavorable equilibrium, which was overcome when the hydrated product was removed by the aldolase protein [95]. It was further shown that the aldolase associates with the DUF35 domain of the *α*-subunit of the heteromeric hydratase complex [95,100]. In *P. stutzeri* Chol1, the aldolase Sal2 is essential for the latter reaction step [84]. During the degradation of cholic acid, a *sal2* deletion mutant accumulated a C_3_ side chain derivative with a double bond and one with a hydroxy group at C17 in minor amounts. This agrees with the notion, that the side chain hydration reaction requires the presence of the aldolase protein to proceed to completion. Like the Sal1 protein, Sal2 and its homologs in *R. jostii* RHA1, *T. curvata*, and *M. tuberculosis* do not harbor the catalytically active cysteine residue known from thiolases. Here, however, the tertiary hydroxy group formed during C_3_ side chain hydration precludes oxidation to a keto group and subsequent thiolytic cleavage of propionyl-CoA.

No homologs of C_3_ side-chain degradation enzymes, including Sal1, FadA5, Sad, or Sal2/Ltp2 were found in *Sphingobium* strain Chol11 and related bile-acid degrading *Sphingomonadaceae*., further supporting an alternative side-chain degradation pathway [23,41,78] (preprint).

A-ring oxidation and side-chain degradation result in the formation of androsta-1,4-diene-3,17-diones (ADDs), which represent key intermediates in bile acid, cholesterol, and testosterone degradation. In the degradation of cholic acid, the removal of the C_3_-side chain leads to 7*α*,12*α*-dihydroxy-androsta-1,4-diene-3,17-dione (12*α*-DHADD, XII). The respective aerobic and anaerobic degradation pathways for ADDs are in large parts identical for bile acid, testosterone, and cholesterol degradation.

### 2.6. Aerobic Cleavage of the Steroid Skeleton via the 9,10-Seco Pathway (Phase 3a)

#### 2.6.1. 9*α*-Hydroxylation: Opening of the B-Ring

Aerobic degradation of ADDs is initiated by hydroxylation at the C9 atom of ring B which is catalyzed by 3-ketosteroid 9*α*-hydroxylases (Figure 5A) [46]. The resulting 3-keto-Δ^1,4^-9*α*-hydroxy intermediate is unstable and reacts in a spontaneous retro-aldol reaction opening ring B between C9 and C10, which is driven by concomitant aromatization of the A-ring [101]. In the degradation of cholic acid, this reaction leads to 3,7,12-trihydroxy-9,10-*seco-*androsta-1,3-5(10)-triene-9,17-dione (THSATD, XIII).

3-ketosteroid 9*α*-hydroxylases have mainly been characterized in Actinobacteria and are monooxygenases composed of a terminal oxygenase and a reductase, which are encoded by *kshA* and *kshB*, respectively [46,102]. The Rieske oxygenase KshA contains a non-heme mononuclear iron and an iron-sulfur cluster and catalyzes substrate 9*α*-hydroxylation. KshB is a ferredoxin reductase, which oxidizes NADH and transfers the electrons through a flavin co-factor and a plant-type iron-sulfur cluster to the iron-sulfur cluster of KshA [103].

Steroid-degrading *Rhodococcus* spp. typically encode several KshA isoenzymes with different affinities towards 3-keto steroid substrates [104]. In *R. rhodochrous* DSM43269 and *R. jostii* RHA1, one distinct *kshA* gene is upregulated during growth with cholic acid and its deletion abolishes cholic acid degradation indicating its essential and specific role in bile acid degradation [28,104,105]. The respective KshA isoenzyme in *R. jostii* RHA11 preferentially catalyzes the hydroxylation of cholic acid degradation intermediates that still carry a C_5_ or C_3_ side chain indicating that side chain and A/B-ring degradation proceed simultaneously in Actinobacteria [105].

In contrast to that, 9*α*-hydroxylation in the bile acid-degrading Proteobacteria *P. stutzeri* Chol1 and *C. testosteroni* TA441 only occurs after complete side chain removal and requires the isomerization of the 12*α*-hydroxy group into a *β*-hydroxy group in 12-hydroxy bile acids [33,37] (see below). In *P. stutzeri* Chol1, the end product of cholic acid side chain degradation is transformed into the respective 9,10-*seco* steroid via an oxygenase-dependent reaction step [19]. A *kshA_Chol1_* deletion mutant strain transforms bile acids to their respective hydroxylated ADDs and shows diminished growth with bile acid substrates [78] (preprint). Additionally, heterologously produced KshAB_Chol1_ hydroxylated a variety of differently hydroxylated ADDs, but no compounds with side chains. In *Sphingobium* sp. strain Chol11, formation of *seco*-steroids from cholic acid was not observed in the medium of wild-type cultures growing with cholic acid, but the strain encodes five homologs of KshA, three of which were detected in a proteome study in cells adapted to steroid substrates. These had low activity towards bile acid degradation intermediates with a Δ^4,6^-structure [23,41,78] (preprint). Interestingly, neither *Sphingobium* sp. strain Chol11 [41,78] (preprint) nor the steroid-degrading *C. tardaugens* NBRC16725 [106] seems to encode distinct homologs of the reductase compound KshB, which might indicate an alternative electron shuttling mechanism in these strains.

#### 2.6.2. Opening and Hydrolysis of the A-Ring

Further degradation of 9,10-*seco* steroids is initiated by reactions that are well known from the aerobic degradation of aromatic compounds [107]. The aromatized A-ring is first hydroxylated at C4 leading to vicinal hydroxy groups as in catechol, which is a central intermediate in the aerobic degradation of aromatic compounds. This hydroxylation is catalyzed by two-component flavin-dependent monooxygenases [47]. In *C. testosteroni,* the enzymes are encoded by *tesA1* and *tesA2* [108]. *R. jostii* RHA1 encodes several isoenzymes of the respective monooxygenase subunits HsaA and HsaB, but only the corresponding genes located in the cholic-acid degradation gene cluster were upregulated during growth with cholic acid [28].

In the next step, the aromatic ring is subject to *meta*-cleavage between C4 and C5 converting the former A-ring into a 2-hydroxy-6-keto-carboxylic acid [97]. This cleavage is catalyzed by TesB in *C. testosteroni* [109] and by HsaC in steroid-degrading Actinobacteria [110,111]. HsaC is a type-I extradiol dioxygenases with a non-heme Fe^2+^ at the catalytic site. The HsaC paralog encoded in the cholic-acid degradation gene cluster of strain RHA1 is upregulated during growth with cholic acid [28].

Further cleavage of a 2-hydroxy-hexa-2,4-dienoate moiety from the opened A-ring leads to the formation of perhydroindane derivatives (3a*α*-*H*-4*α*(3-propanoate)-7a*β*-methylhexahydro-1,5-indandione (HIPs)) consisting of the former C- and D-rings with a C_3_ carboxylic side chain derived from the former B-ring. In cholic acid degradation, this reaction leads to a 7*β*,3′*α*-dihydroxy derivative of HIP (DH-HIP, VIII). The respective hydrolase is encoded by *tesD* in *C. testosteroni* [112], and by *hsaD* in steroid-degrading actinomycetes [111]. HsaD is a *meta*-cleavage product hydrolase of the *α*,*β*-hydrolase superfamily [113,114,115].

The 2-hydroxy-hexa-2,4-dienoate is further degraded via 4-hydroxy-2-oxohexanoate to pyruvate and propionyl-CoA [116]. *tesEFG* and *hsaEFG* encode the enzymes catalyzing the degradation of 2-hydroxy-hexa-2,4-dienoate in *C. testosteroni* and *M. tuberculosis*, respectively [66,111,116,117]. The *hsaEFG* homologs in the bile-acid degradation gene cluster in *R. jostii* RHA1 were upregulated with cholate as substrate.

Homologs of all key enzymes required for the metabolism of the A/B-ring are also found in the cholic acid degradation gene clusters of *P. stutzeri* Chol1, *Sphingobium* sp. strain Chol11 and *Azoarcus* sp. strain Aa7 [23,41,84].

### 2.7. Anaerobic Cleavage of the Steroid Skeleton via the 2,3-Seco Pathway (Phase 3b)

In contrast to the complete anaerobic degradation of sterols and testosterone, which has been reported for a number of denitrifying Proteobacteria [41,118,119,120,121], complete degradation of bile acids in the absence of elemental oxygen has only been reported for *Azoarcus* sp. strain Aa7 [25]. Genomic, biochemical, and physiological analyses suggested that strain Aa7 uses the oxygen-independent 2,3-*seco* pathway for the anaerobic degradation of the steroid nucleus (Figure 5B), which was first described for the anaerobic degradation of cholesterol and testosterone in *Sterolibacterium denitrificans* [50,120,122], *Steroidobacter denitrificans* [119,123] and *Thauera* [121].

The 2,3-*seco* pathway is initiated by a yet unknown enzyme that catalyzes the reduction of the ∆^4^-double bond of the ADD substrate. The next steps are catalyzed by the bifunctional molybdopterin-containing hydratase/dehydrogenase protein complex AtcABC, which adds a water molecule to the Δ^1^-double bond of the A-ring at C1 and subsequently oxidizes the hydroxy group into a keto group [49,124]. Homologs of AtcABC with high similarities to the characterized proteins from *Stl. denitrificans* are encoded in the bile acid degradation gene cluster of strain Aa7 [25]. A so far unknown enzyme catalyzes an oxygen-independent opening of the A-ring resulting in the formation of the 2,3-*seco* intermediate 1,17-dioxo-2,3-*seco*-androstan-3-oic acid (DSAO) [49,123]. A derivative of this compound was also identified in culture supernatants of strain Aa7 growing with cholate under denitrifying conditions [25]. After CoA-activation of this compound, acetyl-CoA is removed via a retro-aldol cleavage [49], before the B-ring is cleaved by so far unknown reactions. This leads to the formation of perhydroindane derivatives (HIPs) as in the aerobic 9,10-*seco* pathway. Thus, the aerobic and anaerobic bile acid degradation pathways converge at the stage of HIP. *Azoarcus* sp. strain Aa7 is able to grow with cholic, chenodeoxycholic, and deoxycholic acid under aerobic and anaerobic conditions, and its genome encodes both the 9,10-*seco*- and the 2,3-*seco* pathway for ADD degradation [25]. Interestingly, this strain requires the activity of the 7*α*-hydroxysteroid dehydratase Hsh2 (see below) to be able to channel 7*α*-hydroxy bile acids into the 2,3-*seco* pathway under anaerobic conditions. A *hsh2* gene deletion mutant accumulated 12*β*-DHADD (VII) as end products of bile acid degradation under anaerobic conditions from the 7*α*-hydroxy bile acids cholic and chenodeoxycholic acid.

### 2.8. C/D-Ring Degradation (Phase 4)

Degradation of rings A and B leads to the formation of perhydroindane derivatives (HIPs) consisting of former rings C and D with a carboxylic C_3_ side chain derived from former ring B. A gene cluster encoding HIP degradation is conserved in steroid-degrading bacteria (Table 1) suggesting a canonical HIP-degradation pathway for cholesterol, testosterone, and bile acids (Figure 6) [24,27,28,32,49,125]. This is further supported by the fact, that genes required for C/D-ring degradation are only found in the cholesterol degradation cluster in sterol and bile acid-degrading Actinobacteria, but not in the respective bile acid degradation cluster [28].

HIP degradation is initiated by CoA-activation of the C_3_-side chain. In *P. putida* DOC21 [35], *C. testosteroni* TA441 [38] and *R. jostii* RHA1 [82], HIP CoA-ligases are encoded by the genes *stdA3*, *fadD3*, and *scdA*, respectively. After activation with CoA, the side chain is further degraded via a *β*-oxidation-like reaction sequence [39]. In bile acid degradation, this reaction sequence can depend on the organism and on the hydroxylation pattern of the bile acid substrate, which is discussed in more detail below. In general, the side chain of HIP-CoA is dehydrogenated by heterotetrameric ACADs similar to the ones involved in steroid side-chain degradation [126]. In *P. stutzeri* Chol1 and *C. testosteroni* TA441, these ACADs are encoded by the genes *scd3A*/*scd3B* and *scdC1/scdC2*, respectively [84,127]. The resulting double bond has been suggested to be hydrated by an enoyl-CoA hydratase, followed by dehydrogenation of the resulting hydroxy group [36,125]. In *C. testosteroni* TA441 the genes *scdD* and *scdE*, encoding an enoyl-CoA hydratase and a 3-hydroxylacyl CoA-dehydrogenase have been shown to be involved in these reactions [39]. Subsequently, an acetyl-CoA moiety is removed from the side chain [125]. This reaction is presumably catalyzed by a 3-ketoacyl-CoA transferase in *C. testosteroni* TA441 encoded by the gene *scdF* [39]. Homologs of these genes are also encoded in the HIP-degradation gene cluster of *P. stutzeri* Chol1 and a respective HIP intermediate with a shortened CoA-activated carboxylic C_1_ side chain has been identified [84].

Further degradation of these compounds has been primarily studied in *Mycobacterium* and *Rhodococcus* strains [125] as well as *C. testosteroni* TA441 [39,128,129,130]. Briefly, a double bond is introduced into former ring C and former ring D is hydrolytically cleaved by an enoyl-CoA hydratase. Subsequently, ring C is opened and the resulting linear bicarboxylic acid is cleaved into propionyl-CoA and succinyl-CoA. Homologs of all key enzymes involved in this degradation are also present in other bile acid-degrading bacteria (Table 1).

### 2.9. Fate of Hydroxy Groups on the Steroid Nucleus

The number, position, and conformation of hydroxy substituents on the steroid nucleus play a crucial role in the bacterial degradation of bile acids. Although *R. jostii* RHA1 can grow with cholic acid, it is not capable of metabolizing other bile acids such as chenodeoxycholic acid or deoxycholic acid [28]. *P. stutzeri* Chol1 grows with cholic, chenodeoxycholic, deoxycholic, and lithocholic acid but it cannot degrade the steroid skeleton of ursodeoxycholic acid (V) which is the 7*β*-epimer of chenodeoxycholic acid [40,84] (Figure 2). Accordingly, degradation of different bile acids requires adaptational reaction steps, depending on the bile acid hydroxylation pattern.

#### 2.9.1. Fate of the 12*α*-Hydroxy Group

**Figure 7:**
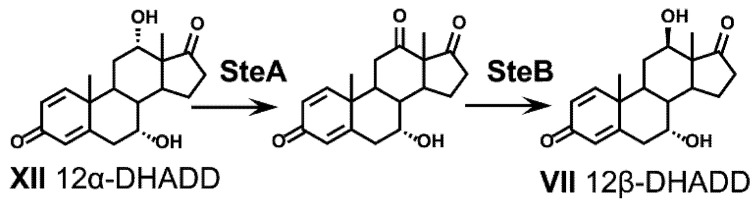
Stereoconversion of the 12-hydroxy group during bile acid degradation. Enzymes were identified in *C. testosteroni* TA441. For bile acids, the respective deprotonated bile salts are depicted.

During degradation of 12*α*-hydroxy bile acids, the 12*α*-hydroxy group is isomerized to the *β*-conformation in *P. stutzeri* Chol1 and *C. testosteroni* TA441 via consecutive oxidation and reduction reactions (Figure 7) [19,37]. In *C. testosteroni*, this inversion is catalyzed by the dehydrogenases SteA and SteB and has been proposed to be indispensable for the following 9*α*-hydroxylation step opening the B-ring [37]. In *P. stutzeri* Chol1, the 12*α*-hydroxy group of 12*α*-DHADD (XII) is oxidized with NAD^+^ and the resulting 12-keto intermediate is subsequently reduced to 12*β*-DHADD (VII) with NADPH as electron donor [33]. After removal of the A-ring, the former 12*β*-hydroxy group is removed by a two-step reductive dehydroxylation catalyzed by a dehydratase (Hsh1) and an oxidoreductase (Sor1) in strain Chol1 (Figure 6) [84]. Homologs of *steA*, *steB*, *sor1*, and *hsh1* are found in the steroid degradation gene clusters of *C. testosteroni*, *Sphingobium* sp. strain Chol11 and *Azoarcus* sp. strain Aa7 in distinct sub-clusters with similar gene syntenies [23,84], suggesting that the isomerization and subsequent reductive dehydroxylation of the 12*α*-hydroxy group are characteristic features in bile acid-degrading Proteobacteria. The formation of a HIP derivative without a hydroxy group at former C12 during cholic acid degradation [43] suggests that this hydroxy group is also removed prior to HIP degradation in *R. jostii* RHA1, which was hypothesized to be catalyzed by a putative enoyl-CoA hydratase [125].

#### 2.9.2. Fate of the 7*α*-Hydroxy Group

Transformation or degradation reactions of the 7*α*-hydroxy group of cholic and chenodeoxycholic acid differ widely among bile acid-degrading bacteria. In *P. stutzeri* Chol1, and presumably *R. jostii* RHA1, the 7*α*-hydroxy group is maintained up to the level of HIP degradation [43,84]. During HIP degradation, a hydroxy group in *β*-position is required for the removal of the carboxylic C_3_ side chain (see above, Figure 6). *P. stutzeri* Chol1 mutant strains lacking the genes encoding the HIP-CoA ACAD system, which introduces a double bond into the HIP side chain prior to hydroxylation, showed no phenotype growing with the 7*α*-hydroxy bile acids cholic and chenodeoxycholic acid compared to the wildtype [84]. In contrast, these mutants accumulated a HIP derivative from deoxycholic and lithocholic acid. This suggested that dehydrogenation and hydration of the HIP side chain are not required during degradation of 7*α*-hydroxy bile acids in this strain and that the degradation pathways of cholic, deoxycholic, chenodeoxycholic, and lithocholic acid converge at a central HIP-derivative carrying a hydroxy group in the C_3_ side chain [84].

In contrast, recent studies have suggested that the 7*α*-hydroxy group of cholic acid is removed at the stage of HIP degradation in *C. testosteroni* TA441. Gene deletion mutants lacking the *scdD* (enoyl-CoA hydratase), *scdE* (enoyl-CoA hydratase), and *scdF* (*3*-ketoacyl-CoA transferase) genes, which were shown to be involved in the removal of the C_3_ HIP side chain, accumulated a HIP derivative with a double bond in the C_3_ side chain from different steroid substrates, including 7*α*-hydroxy bile acids [36,39]. The authors speculated that the 7*α*-hydroxy-group is removed by dehydration after CoA-activation of the HIP side chain and that subsequent hydration of the resulting double bond might result in a stereo-inversion of the hydroxy group.

#### 2.9.3. Dehydratation of 7-Hydroxy Groups during Bile Acid Degradation via Δ^4,6^-3-Keto Intermediates

**Figure 8:**
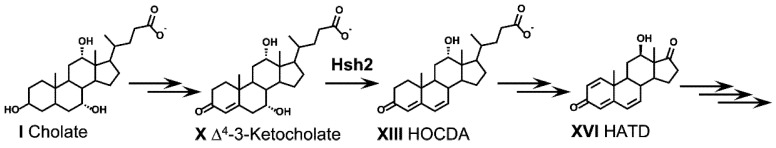
Degradation of bile acids via the Δ^4,6^-variant of the 9,10-*seco* pathway as found, e.g., in *Sphingobium* sp. strain Chol11. For bile acids, the respective deprotonated bile salts are depicted. Abbreviations: HATD, 12*β*-Hydroxy-androsta-1,4,6-triene-3,17-dione.

The *α*-Proteobacteria *Sphingobium* sp. strain Chol11, *Sphingobium herbicidovorans* MH, *Novosphingobium aromaticivorans* F199, and *C. tardaugens* NBRC16725 initiate the degradation of cholic acid and other 7-hydroxy bile acids via an alternative route, which had been designated the ∆^4,6^-variant [23,40] (Figure 8). Additionally, the Actinobacterium *Dietzia* sp. strain Chol2 uses this variant [18]. In these organisms, the 7-hydroxy group is removed from the intact bile acid molecule after partial oxidation of the A-ring via an enzymatic dehydration reaction, leading to the formation of a double bond between C6 and C7 (∆^6^). In *Sphingobium* sp. strain Chol11, dehydration of the 7*α*-hydroxy group is catalyzed by the hydroxysteroid dehydratase Hsh2 with 3-keto-Δ^4^ intermediates as physiological substrates [40], yielding intermediates with a 3-keto-Δ^4,6^-diene structure, such as 12-hydroxy-3-oxo-4,6-choldienoic acid (HOCDA, XIV). As mentioned above, this reaction is required by *Azoarcus* sp. strain Aa7 to be able to degrade 7*α*-hydroxy bile acids via the anaerobic 2,3-*seco* pathway [25]. In contrast, Hsh2 is not essential for the growth of *Sphingobium* sp. strain Chol11 with cholic acid or chenodeoxycholic acid but is necessary for maximum growth efficiency with these 7*α*-hydroxysteroids. The resulting Δ^6^-double bond is not reduced or rehydrated prior to 9*α*-hydroxylation and subsequent degradation of the bile acid nucleus [18,40]. *Sphingobium* sp. strain Chol11 harbors at least one more hydroxysteroid dehydratase specific for the 7*β*-hydroxy group of ursodeoxycholic acid [40]. In supernatants of cholic acid-grown cultures of this strain, 12*β*-hydroxy-androsta-1,4,6-triene-3,17-dione (HATD, XVI) occurs as a degradation intermediate [18]. This ADD-derivative with an additional ∆^6^-double bond indicates that side-chain degradation is the next metabolic step in the degradation of 3-keto-Δ^4,6^-diene intermediates. The first results indicate that the further degradation of HATD proceeds via a 9,10-*seco*-steroid [23,78] (preprint). The Δ^4,6^ degradation pathway variant for 7*α*-hydroxy bile acids is currently under investigation with a special focus on the above-mentioned differences in side-chain degradation.

## 3. Resistance Mechanisms against the Toxic Effects of Bile Acids

Bile acids and their derivatives are highly toxic compounds for bacterial cells. Due to their amphiphilic character, bile acids lead to an increase in membrane permeability and may cause cell lysis [131,132]. In the bacterial cytoplasm, bile acids can cause DNA damage, protein-unfolding, and disulfide stress [133,134].

In the small intestine, bile acids have been shown to possess an important controlling effect for the bacterial population due to their antimicrobial properties [9]. Intestinal bacteria, facing up to 20 mM bile-acid concentrations in the duodenum [54], have evolved multiple resistance mechanisms against the toxic effects of bile acids [132,135]: The outer membrane of Gram-negative bacteria acts as a diffusion barrier and constitutes the first protection against bile acid toxicity [136,137]. Additionally, many intestinal bacteria have energy-dependent efflux systems constituting effective protection by means of avoiding the accumulation of bile acids in the cytoplasm [54,132,135,138].

Similarly, bacteria using bile acids as growth substrates must actively protect themselves against the toxic effects of bile acids. However, for metabolism and energy conservation, these carbon- and energy-rich but lethal compounds must be taken up into the cytoplasm. Moreover, degradation intermediates such as ADD and 9,10-*seco* metabolites can exhibit additional toxicity in the cell [19,139,140]. Thus, bile acid-degrading bacteria must possess precisely regulated protection mechanisms, which keep the intracellular concentration of toxic bile acids and their degradation intermediates below a critical level, while providing sufficient intracellular bile acid substrate concentrations for metabolism and energy conservation. Presumably, part of the energy generated during bile acid metabolism must be invested into protection mechanisms.

To that effect, degradation itself poses an effective detoxification mechanism. Especially the first A-ring oxidation steps presumably produce less toxic compounds as the removal of one hydroxy group from the hydrophilic side of the molecule probably decreases the detergent character. As a protection mechanism, degradation should be fast and tightly regulated to prevent overflow at any phase. Interestingly, the 5*β*-Δ^4^-KSTD from *Sphingobium* sp. strain Chol11 involved in A-ring oxidation is a very fast enzyme that showed strong substrate inhibition [42]. This results in a fast transformation of 3-keto bile acids to their less toxic Δ^4^ derivatives, while an overflow of the subsequent pathway including slower enzymes [40,41] is prevented.

Additionally, it has been speculated that efflux-pump-mediated excretion of bile acids and some degradation intermediates might contribute to the control of the intracellular concentration [31,43]. The susceptibility of *P. stutzeri* Chol1 to cholic acid increases in the presence of the uncoupling agent carbonyl cyanide *m*-chlorophenylhydrazone (CCCP), indicating that an intact proton-motive-force contributes to the protection against bile acids [19]. These findings support the hypothesis that the efflux of bile acids is a crucial defense mechanism in bile acid-degrading bacteria. The steroid degradation gene cluster of *P. stutzeri* Chol1 contains a gene encoding a Resistance-Nodulation-Division type (RND) transporter, which is a candidate for such an efflux system. In *R. jostii* RHA1 two transporters belonging to the ATP-binding cassette (ABC) family and major facilitator superfamily (MFS) are essential for the re-uptake of transiently excreted and potentially toxic cholic-acid degradation intermediates [43]. Further research is required to substantiate the role of exporters in bile-acid metabolism.

## 4. Regulation of Bile-Acid Degradation

The structural complexity and the molecular size of bile acids require more than thirty biochemical steps for their complete degradation to CO_2_, suggesting that bile acid degradation has to be tightly regulated. In addition, adaptation to bile acid toxicity presumably requires further regulatory mechanisms controlling the uptake and export of substrates and degradation intermediates. In *P. stutzeri* Chol1 [26], *Sphingobium* sp. strain Chol11 [23], and *Dietzia* sp. strain Chol2 (unpublished data), complete cholic acid degradation is not induced in cells grown with non-steroidal substrates, indicating regulatory mechanisms for bile-acid degradation in these strains. However, the knowledge about the regulation of bile acid metabolism is limited. For *Sphingobium* sp. strain Chol11, proteome analyses clearly showed that enzymes encoded in several gene clusters were significantly more abundant when the strain was grown with bile acids, indicating regulation of these clusters in response to the presence of bile acids [23].

In *C. testosteroni*, TeiR and HsdR are positive regulators of steroid metabolism [141,142]. TeiR localizes at one of the cell poles, enabling steroid sensing and motility towards steroid substrates. The binding of testosterone and some other steroids to TeiR activates a kinase-dependent signaling mechanism. The LysR-type transcriptional regulator HsdR activates the transcription of *hsdA* encoding a 3*α*-hydroxysteroid-dehydrogenase [142]. Two genes encoding putative TeiR-like transcriptional regulators are also found in the steroid degradation gene cluster of *P. stutzeri* Chol1. Deletion of one of these genes (*C211_RS11295*) led to a strong delay of the onset of cholic acid degradation and growth, indicating a regulatory function of the respective protein during bile acid degradation (unpublished data). However, distinct regulatory mechanisms are still largely unknown for bile acid-degrading Proteobacteria.

In *R. jostii* RHA1, the expression of cholic acid degradation genes is upregulated when growing with cholic acid, but not in the presence of cholesterol or pyruvate [28,90]. This indicates that distinct regulatory mechanisms control the expression of the different steroid-degradation gene clusters in this strain. In *R. jostii* RHA1 and other steroid-degrading Actinobacteria, the TetR-type transcriptional repressors KstR1 and KstR2 have been shown to control sterol metabolism [143]. KstR1 regulates the sterol side-chain and A/B ring degradation, which are induced by the cholesterol-degradation intermediate 3-oxo-4-cholestenoic acid [144]. KstR2 regulates HIP degradation and KstR2 repression is relieved by HIP-CoA-esters and a lactone derivative of HIP [144,145]. Homologs of KstR1 and KstR2 are not present in bile acid-degrading Proteobacteria, but a KstR homolog is encoded in the cholic-acid degradation gene cluster of *R. jostii* RHA1.

## 5. Potential Ecological Effects of Bacterial Bile-Acid Degradation

Besides their function as detergents in digestion, bile acids act as regulatory compounds [3,146,147] and also signaling compounds between different organisms, e.g., in aquatic vertebrates [15,16]. As such, distinctive bile acids and bile alcohols function as migratory hormones in aquatic vertebrates such as sea lampreys [15,148,149,150,151]. The bile acid 5*α*-cyprinol sulfate that is excreted by fish induces a predator avoidance behavior in *Daphnia* called diel vertical migration and thus acts as a chemical cue for *Daphnia* to sense the presence of predators [152]. Additionally, it was recently reported that bile acids are natural ligands of the mouse accessory olfactory system [153] indicating that they may also have a signaling function among terrestrial vertebrates.

Therefore, bacterial bile acid degradation in these habitats may interfere with those signaling systems via the removal or transformation of signaling compounds. The degradation of these signal compounds could also be very important, e.g., in the case of *Daphnia* where bile acids indicate the presence of predators, a system that only works if bile acids are removed again. Furthermore, interference of bile acid degradation intermediates with signaling pathways might be possible, especially as bile acids and alcohols with a 3-keto-Δ^4,6^ structure similar to HOCDA (XIV) are pheromones of the sea lamprey *Petromyzon marinus* [149,154].

Besides possible effects on vertebrates, bile acid degradation might also have an impact on invertebrates such as the soil nematode *Caenorhabditis elegans* [155,156]. *C. elegans* possesses at least 284 genes for nuclear hormone receptors (NHR), which bind steroid hormones and other ligands, in contrast to only 48 genes coding for NHR in humans [157]. Dafachronic acids, which are similar to bile acids, are important NHR-binding steroid hormones in *C. elegans*, regulating development [158,159]. Bacterial degradation or transformation of dafachronic acids could interfere with this mechanism.

Steroid intermediates such as ADDs accumulate transiently in the extracellular environment during bacterial degradation of bile acids under laboratory conditions [18,19,31,43] and during degradation of chenodeoxycholic acid in soil samples [12]. In analogy, bacterial bile acid transformation products may also occur in aquatic and terrestrial environments with a high input of bile acids such as manured agricultural soils, making them available to other bacteria and eukaryotic organisms. A transient accumulation of ADDs has been shown [160] and calculated in models [161] for C_19_ steroids that are produced during the microbial degradation of sterol compounds from manure fertilizer.

Thus, these compounds may have ecological effects as some of the degradation intermediates are very similar to steroid hormones. For example, the bacterial degradation of lithocholic acid produces ADD [84], which has androgenic effects on mammals, similar to testosterone [162,163]. In addition, differently hydroxylated ADDs and ADs are exported as prominent intermediates during the degradation of other bile acids [18,19,31,66]. Although only little information is available regarding the endocrine effects of hydroxylated ADDs and ADs, they might also have androgenic potential, since exogenous steroids with similar structures to testosterone affect the androgen signaling pathways in vertebrates [164,165]. Similarly, endocrine effects of bacterial sterol degradation on fish were suggested: masculinization of fish populations in rivers receiving paper-mill effluents [166,167,168] was traced back to the bacterial transformation of plant-derived sterols into androgenic steroids [169,170,171].

During the degradation of chenodeoxycholic acid (II), 7*α*-OH ADD is produced, which was shown to have adverse effects on the nematode *Caenorhabditis elegans* [12]. 7*α*-OH ADD decreased the reproduction and development and induced behavioral changes of *C. elegans* when added to the medium. A deletion mutant lacking the putative testosterone receptor NHR-69 did not display this phenotype, suggesting that 7*α*-OH ADD effects in *C. elegans* are mediated via this receptor NHR-69. Thus, the remarkably high number of NHR in *C. elegans* could be important for environmental signaling via steroid compounds potentially including bacterial bile-acid degradation intermediates [172,173,174].

However, for assessing potential endocrine effects of steroids originating from bacterial bile acid degradation on soil and water fauna, especially in areas with a high input of manure, further studies are necessary.

The transient extracellular accumulation of steroid degradation intermediates could also lead to cross-feeding processes between bile acid-degrading bacteria in soil and water. Given the abundance of bile acid-degrading bacteria, these bacteria might not only compete for these energy-rich substrates but also for their degradation intermediates. Cross feeding has been studied with *P. stutzeri* Chol1 and *Sphingobium* sp. strain Chol11. While *P. stutzeri* Chol1 degrades 7*α*-hydroxy bile acids, such as cholic acid and chenodeoxycholic acid, via intermediates with a 3-keto-Δ^1,4^ structure of the steroid (e.g., VI), *Sphingobium* sp. strain Chol11 degrades these compounds via intermediates with a 3-keto-Δ^4,6^ structure (XIV) [18,40]. However, the latter strain is also able to use steroids with a 3-keto-Δ^1,4^ structure for growth. In contrast to that, *P. stutzeri* cannot grow with Δ^4,6^-3-keto compounds formed by *Sphingobium* sp. strain Chol11 and transforms them into dead-end metabolites that accumulate in the culture supernatant. Apparently, *Sphingobium* sp. strain Chol11 has a broader metabolic repertoire for the utilization of bile acids, which could be a selective advantage in competing for these substrates in natural habitats [40]. Additionally, the conversion of bile acids into steroids, which are not bioavailable to competitors, could also serve as a strategy of excluding competition. The fact that cross-feeding processes could occur as well as the observation that such cross-feeding may create novel steroid compounds with potential endocrine effects reveals the complexity and the impact that bacterial bile-acid degradation might have in natural habitats.

## 6. Applied Aspects of Bacterial Bile-Acid Degradation

Plant-based phytosterols such as stigmasterol, campesterol, and diosgenin are the major raw materials for the steroid industry today [175]. These starting materials are microbially transformed into a few key products, e.g., ADD and 20-carboxy-pregna-4-en-3-one, which are subsequently modified to high-value drugs such as contraceptives and corticosteroids. However, biotechnological processes are also required for the production of bile acid-derived drugs [176]. Ursodeoxycholic acid (V) inhibits the proliferation of colon cancer cells and is used as an agent for the treatment of primary sclerosing cholangitis and for the dissolution of gallstones [177,178,179]. The chemical synthesis of ursodeoxycholic acid from cholic acid is accomplished by seven chemical reactions with a yield of about 30%. Using a bacterial whole-cell biotransformation process combined with these chemical steps has been reported to increase the yield to 95% [180].

Probiotic bacteria could be used to increase bacterial bile-acid deconjugation in the gut, which would decrease the re-absorption of bile acids in the enterohepatic cycle and consequently increase bile-acid production from cholesterol leading to higher cholesterol consumption in the body [181,182,183].

## 7. Conclusions and Future Perspectives

The bacterial degradation of bile acids has numerous relevant aspects for fundamental and applied research. Thirty-nine years after the seminal review by Hayakawa about bacterial bile acid degradation [48] the genes and enzymes for many of the postulated metabolic pathways have been characterized. In addition, novel and unexpected reactions for the breakdown of bile acids have been discovered indicating alternative pathways, which are currently being analyzed. Here, the elucidation of side-chain degradation by the *Sphingomonadaceae* is a key aspect. The understanding of the metabolic pathways for bile acid degradation is the prerequisite for addressing the potential ecological effects of the hormone-like metabolites that arise as intermediates during the breakdown of bile acids in soil and water. As these metabolic pathways comprise numerous reaction steps and as the parent molecules, as well as some degradation metabolites, have toxic effects, bile acid degradation is a physiological challenge for bacteria that will require complex regulatory networks. Finally, the ongoing discovery of novel physiological functions of bile acids in vertebrates [2] indicates that the targeted biotechnological production of novel bile acid-derivatives with engineered bacterial strains might have pharmacological potential.

## References

[1] Hofmann A.F., Hagey L.R., Krasowski M.D. (2010). Bile salts of vertebrates: Structural variation and possible evolutionary significance. J. Lipid Res..

[2] Vítek L., Haluzík M. (2016). The role of bile acids in metabolic regulation. J. Endocrinol..

[3] Hylemon P.B., Zhou H., Pandak W.M., Ren S., Gil G., Dent P. (2009). Bile acids as regulatory molecules. J. Lipid Res..

[4] Ridlon J.M., Kang D.-J.J., Hylemon P.B. (2006). Bile salt biotransformations by human intestinal bacteria. J. Lipid Res..

[5] Funabashi M., Grove T.L., Wang M., Varma Y., McFadden M.E., Brown L.C., Guo C., Higginbottom S., Almo S.C., Fischbach M.A. (2020). A metabolic pathway for bile acid dehydroxylation by the gut microbiome. Nature.

[6] Ridlon J.M. (2020). Conceptualizing the vertebrate sterolbiome. Appl. Environ. Microbiol..

[7] Doden H.L., Ridlon J.M. (2021). Microbial hydroxysteroid dehydrogenases: From alpha to omega. Microorganisms.

[8] Winston J.A., Theriot C.M. (2020). Diversification of host bile acids by members of the gut microbiota. Gut Microbes.

[9] Ridlon J., Kang D., Hylemon P., Bajaj J. (2014). Bile acids and the gut microbiome. Curr. Opin. Gastroenterol..

[10] Kriaa A., Bourgin M., Potiron A., Mkaouar H., Jablaoui A., Gérard P., Maguin E., Rhimi M. (2019). Microbial impact on cholesterol and bile acid metabolism: Current status and future prospects. J. Lipid Res..

[11] Tyagi P., Edwards D.R., Coyne M.S. (2008). Use of sterol and bile acid biomarkers to identify domesticated animal sources of fecal pollution. Water. Air. Soil Pollut..

[12] Mendelski M.N., Dölling R., Feller F.M., Hoffmann D., Ramos Fangmeier L., Ludwig K.C., Yücel O., Mährlein A., Paul R.J., Philipp B. (2019). Steroids originating from bacterial bile acid degradation affect *Caenorhabditis elegans* and indicate potential risks for the fauna of manured soils. Sci. Rep..

[13] Tyagi P., Edwards D.R., Coyne M.S. (2009). Fecal sterol and bile acid biomarkers: Runoff concentrations in animal waste-amended pastures. Water. Air. Soil Pollut..

[14] Elhmmali M.M., Roberts D.J., Evershed R.P. (2000). Combined analysis of bile acids and sterols/stanols from riverine particulates to assess sewage discharges and other fecal sources. Environ. Sci. Technol..

[15] Buchinger T.J., Li W., Johnson N.S. (2014). Bile salts as semiochemicals in fish. Chem. Senses.

[16] Meredith T.L., Caprio J., Kajiura S.M. (2012). Sensitivity and specificity of the olfactory epithelia of two elasmobranch species to bile salts. J. Exp. Biol..

[17] Merino E., Barrientos A., Rodríguez J., Naharro G., Luengo J.M., Olivera E.R. (2013). Isolation of cholesterol- and deoxycholate-degrading bacteria from soil samples: Evidence of a common pathway. Appl. Microbiol. Biotechnol..

[18] Holert J., Yücel O., Suvekbala V., Kulić Ž., Möller H., Philipp B. (2014). Evidence of distinct pathways for bacterial degradation of the steroid compound cholate suggests the potential for metabolic interactions by interspecies cross-feeding. Environ. Microbiol..

[19] Philipp B., Erdbrink H., Suter M.J.F., Schink B. (2006). Degradation of and sensitivity to cholate in *Pseudomonas* sp. strain Chol1. Arch. Microbiol..

[20] Holert J., Cardenas E., Bergstrand L.H., Zaikova E., Hahn A.S., Hallam S.J., Mohn W.W. (2018). Metagenomes reveal global distribution of bacterial steroid catabolism in natural, engineered, and host environments. mBio.

[21] Lengeler J., Drews G., Schlegel H., Lengeler J.W., Drews G., Schlegel H.G. (2009). Biology of the Prokaryotes.

[22] Bull I.D., Lockheart M.J., Elhmmali M.M., Roberts D.J., Evershed R.P. (2002). The origin of faeces by means of biomarker detection. Environ. Int..

[23] Feller F.M., Wöhlbrand L., Holert J., Schnaars V., Mohn W.W., Rabus R., Philipp B. (2021). Proteome, bioinformatic and functional analyses reveal a distinct and conserved metabolic pathway for bile salt degradation in the *Sphingomonadaceae*. Appl. Environ. Microbiol..

[24] Horinouchi M., Hayashi T., Kudo T. (2012). Steroid degradation in *Comamonas testosteroni*. J. Steroid Biochem. Mol. Biol..

[25] Yücel O., Borgert S.R., Poehlein A., Niermann K., Philipp B. (2019). The 7*α*-hydroxysteroid dehydratase Hsh2 is essential for anaerobic degradation of the steroid skeleton of 7*α*-hydroxyl bile salts in the novel denitrifying bacterium *Azoarcus* sp. strain Aa7. Environ. Microbiol..

[26] Birkenmaier A., Holert J., Erdbrink H., Möller H.M., Friemel A., Schönenberger R., Suter M.J.F., Klebensberger J., Philipp B. (2007). Biochemical and genetic investigation of initial reactions in aerobic degradation of the bile acid cholate in *Pseudomonas* sp. strain Chol1. J. Bacteriol..

[27] Bergstrand L.H., Cardenas E., Holert J., van Hamme J.D., Mohn W.W. (2016). Delineation of steroid-degrading microorganisms through comparative genomic analysis. mBio.

[28] Mohn W.W., Wilbrink M.H., Casabon I., Stewart G.R., Liu J., van der Geize R., Eltis L.D. (2012). Gene cluster encoding cholate catabolism in *Rhodococcus* spp.. J. Bacteriol..

[29] Guevara G., de las Heras L.F., Perera J., Llorens J.M.N. (2017). Functional characterization of 3-ketosteroid 9*α*-hydroxylases in *Rhodococcus ruber* strain chol-4. J. Steroid Biochem. Mol. Biol..

[30] Shtratnikova V.Y., Schelkunov M.I., Fokina V.V., Bragin E.Y., Lobastova T.G., Shutov A.A., Kazantsev A.V., Donova M. (2020). V Genome-wide transcriptome profiling provides insight on cholesterol and lithocholate degradation mechanisms in *Nocardioides simplex* VKM Ac-2033D. Genes.

[31] Philipp B. (2011). Bacterial degradation of bile salts. Appl. Microbiol. Biotechnol..

[32] Olivera E.R., Luengo J.M. (2019). Steroids as environmental compounds recalcitrant to degradation: Genetic mechanisms of bacterial biodegradation pathways. Genes.

[33] Holert J., Kulić Ž., Yücel O., Suvekbala V., Suter M.J.F., Möller H.M., Philipp B. (2013). Degradation of the acyl side chain of the steroid compound cholate in *Pseudomonas* sp. strain Chol1 proceeds via an aldehyde intermediate. J. Bacteriol..

[34] Holert J., Jagmann N., Philipp B. (2013). The essential function of genes for a hydratase and an aldehyde dehydrogenase for growth of *Pseudomonas* sp. strain Chol1 with the steroid compound cholate indicates an aldolytic reaction step for deacetylation of the side chain. J. Bacteriol..

[35] Barrientos Á., Merino E., Casabon I., Rodríguez J., Crowe A.M., Holert J., Philipp B., Eltis L.D., Olivera E.R., Luengo J.M. (2015). Functional analyses of three acyl-CoA synthetases involved in bile acid degradation in *Pseudomonas putida* DOC21. Environ. Microbiol..

[36] Horinouchi M., Hayashi T., Koshino H., Malon M., Hirota H., Kudo T. (2014). Identification of 9*α*-hydroxy-17-oxo-1,2,3,4,10,19-hexanorandrost-6- en-5-oic acid and *β*-oxidation products of the C-17 side chain in cholic acid degradation by *Comamonas testosteroni* TA441. J. Steroid Biochem. Mol. Biol..

[37] Horinouchi M., Hayashi T., Koshino H., Malon M., Yamamoto T., Kudo T. (2008). Identification of genes involved in inversion of stereochemistry of a C-12 hydroxyl group in the catabolism of cholic acid by *Comamonas testosteroni* TA441. J. Bacteriol..

[38] Horinouchi M., Hayashi T., Koshino H., Kudo T. (2006). ORF18-disrupted mutant of *Comamonas testosteroni* TA441 accumulates significant amounts of 9,17-dioxo-1,2,3,4,10,19-hexanorandrostan-5-oic acid and its derivatives after incubation with steroids. J. Steroid Biochem. Mol. Biol..

[39] Horinouchi M., Koshino H., Malon M., Hirota H., Hayashi T. (2019). Steroid degradation in *Comamonas testosteroni* TA441: Identification of the entire *β*-oxidation cycle of the cleaved B ring. Appl. Environ. Microbiol..

[40] Yücel O., Drees S., Jagmann N., Patschkowski T., Philipp B. (2016). An unexplored pathway for degradation of cholate requires a 7*α*-hydroxysteroid dehydratase and contributes to a broad metabolic repertoire for the utilization of bile salts in *Novosphingobium* sp. strain Chol11. Environ. Microbiol..

[41] Yücel O., Holert J., Ludwig K.C., Thierbach S., Philipp B. (2018). A novel steroidcoenzyme A ligase from *Novosphingobium* sp. strain Chol11 is essential for an alternative degradation pathway for bile salts. Appl. Environ. Microbiol..

[42] Feller F.M., Marke G., Drees S.L., Wöhlbrand L., Rabus R., Philipp B. (2021). Substrate inhibition of 5*β*-Δ^4^-3-ketosteroid dehydrogenase in *Sphingobium* sp. strain Chol11 acts as circuit breaker during growth with toxic bile salts. Front. Microbiol..

[43] Swain K., Casabon I., Eltis L.D., Mohn W.W. (2012). Two transporters essential for reassimilation of novel cholate metabolites by *Rhodococcus jostii* RHA1. J. Bacteriol..

[44] Wipperman M.F., Sampson N.S., Thomas S.T., Thomas S.T. (2014). Pathogen roid rage: Cholesterol utilization by *Mycobacterium tuberculosis*. Crit. Rev. Biochem. Mol. Biol..

[45] Galán B., García-Fernández J., Felpeto-Santero C., Fernández-Cabezón L., García J.L., Rojo F. (2017). Bacterial metabolism of steroids. Aerobic Utilization of Hydrocarbons, Oils and Lipids.

[46] Petrusma M., Van Der Geize R., Dijkhuizen L. (2014). 3-Ketosteroid 9*α*-hydroxylase enzymes: Rieske non-heme monooxygenases essential for bacterial steroid degradation. Antonie Leeuwenhoek.

[47] Dresen C., Lin L.Y.C., D’Angelo I., Tocheva E.I., Strynadka N., Eltis L.D. (2010). A flavin-dependent monooxygenase from *Mycobacterium tuberculosis* involved in cholesterol catabolism. J. Biol. Chem..

[48] Hayakawa S. (1982). Microbial transformation of bile acids. A unified scheme for bile acid degradation, and hydroxylation of bile acids. Z. Allg. Mikrobiol..

[49] Warnke M., Jacoby C., Jung T., Agne M., Mergelsberg M., Starke R., Jehmlich N., von Bergen M., Richnow H.-H., Brüls T. (2017). A patchwork pathway for oxygenase-independent degradation of side chain containing steroids. Environ. Microbiol..

[50] Wang P.H., Lee T.H., Ismail W., Tsai C.Y., Lin C.W., Tsai Y.W., Chiang Y.R. (2013). An oxygenase-independent cholesterol catabolic pathway operates under oxic conditions. PLoS ONE.

[51] Holert J., Alam I., Larsen M., Antunes A., Bajic V.B., Stingl U., Philipp B. (2013). Genome sequence of *Pseudomonas* sp. strain Chol1, a model organism for the degradation of bile salts and other steroid compounds. Genome Announc..

[52] Horinouchi M., Kurita T., Yamamoto T., Hatori E., Hayashi T., Kudo T., Hirosawa W.-S. (2004). Steroid degradation gene cluster of *Comamonas testosteroni* consisting of 18 putative genes from *meta*-cleavage enzyme gene *tesB* to regulator gene *tesR*. Biochem. Biophys. Res. Commun..

[53] Cabral D.J., Small D.M., Lilly H.S., Hamilton J.A. (1987). Transbilayer movement of bile acids in model membranes. Biochemistry.

[54] Thanassi D.G., Cheng L.W., Nikaido H. (1997). Active efflux of bile salts by *Escherichia coli*. J. Bacteriol..

[55] Mallonee D.H., Hylemon P.B. (1996). Sequencing and expression of a gene encoding a bile acid transporter from *Eubacterium* sp. strain VPI 12708. J. Bacteriol..

[56] Ibero J., Galán B., Rivero-Buceta V., García J.L. (2020). Unraveling the 17*β*-estradiol degradation pathway in *Novosphingobium tardaugens* NBRC 16725. Front. Microbiol..

[57] Somalinga V., Mohn W.W. (2013). *Rhodococcus jostii* porin A (RjpA) functions in cholate uptake. Appl. Environ. Microbiol..

[58] Mohn W.W., Van Der Geize R., Stewart G.R., Okamoto S., Liu J., Dijkhuizen L., Eltis L.D. (2008). The actinobacterial *mce4* locus encodes a steroid transporter. J. Biol. Chem..

[59] Pandey A.K., Sassetti C.M. (2008). Mycobacterial persistence requires the utilization of host cholesterol. Proc. Natl. Acad. Sci. USA.

[60] Oppermann U.C.T., Maser E. (1996). Characterization of a 3*α*-hydroxysteroid dehydrogenase/carbonyl reductase from the Gram-negative bacterium *Comamonas testosteroni*. Eur. J. Biochem..

[61] Maser E., Xiong G., Grimm C., Ficner R., Reuter K. (2001). 3*α*-hydroxysteroid dehydrogenase/carbonyl reductase from *Comamonas testosteroni*: Biological significance, three-dimensional structure and gene regulation. Chem. Biol. Interact..

[62] Kisiela M., Skarka A., Ebert B., Maser E. (2012). Hydroxysteroid dehydrogenases (HSDs) in bacteria—A bioinformatic perspective. J. Steroid Biochem. Mol. Biol..

[63] Chen J., Gao X., Hong L., Ma L., Li Y. (2015). Expression, purification and functional characterization of a novel 3*α*-hydroxysteroid dehydrogenase from *Pseudomonas aeruginosa*. Protein Expr. Purif..

[64] Davidson S.J., Talalay P. (1966). Purification and mechanism of action of a steroid Δ^4^-5-*β*-dehydrogenase. J. Biol. Chem..

[65] Bar-Even A., Noor E., Savir Y., Liebermeister W., Davidi D., Tawfik D.S., Milo R. (2011). The moderately efficient enzyme: Evolutionary and physicochemical trends shaping enzyme parameters. Biochemistry.

[66] Horinouchi M., Hayashi T., Yamamoto T., Kudo T. (2003). A new bacterial steroid degradation gene cluster in *Comamonas testosteroni* TA441 which consists of aromatic-compound degradation genes for *seco*-steroids and 3-ketosteroid dehydrogenase genes. Appl. Environ. Microbiol..

[67] van Oosterwijk N., Knol J., Dijkhuizen L., Van Der Geize R., Dijkstra B.W. (2012). Structure and catalytic mechanism of 3-ketosteroid-Δ^4^-(5*α*)-dehydrogenase from *Rhodococcus jostii* RHA1 genome. J. Biol. Chem..

[68] Dijkstra B.W., Van Oosterwijk N., Rohman A. (2016). Structure and catalytic mechanism of 3-ketosteroid dehydrogenases. Procedia Chem..

[69] Rohman A., Dijkstra B.W. (2021). Application of microbial 3-ketosteroid Δ^1^-dehydrogenases in biotechnology. Biotechnol. Adv..

[70] Olivera E.R., de la Torre M., Barrientos Á., Luengo J.M. (2018). Steroid catabolism in bacteria: Genetic and functional analyses of *stdH* and *stdJ* in *Pseudomonas putida* DOC21. Can. J. Biotechnol..

[71] Florin C., Kohler T., Grandguillot M., Plesiat P. (1996). *Comamonas testosteroni* 3-ketosteroid-Δ^4^-5*α*-dehydrogenase: Gene and protein characterization. J. Bacteriol.

[72] Knol J., Bodewits K., Hessels G.I., Dijkhuizen L., van der Geize R. (2008). 3-Keto-5*α*-steroid Δ^1^-dehydrogenase from *Rhodococcus erythropolis* SQ1 and its orthologue in *Mycobacterium tuberculosis* H37Rv are highly specific enzymes that function in cholesterol catabolism. Biochem. J..

[73] Zhang Q., Ren Y., He J., Cheng S., Yuan J., Ge F., Li W., Zhang Y., Xie G. (2015). Multiplicity of 3-ketosteroid Δ^1^-dehydrogenase enzymes in *Gordonia neofelifaecis* NRRL B-59395 with preferences for different steroids. Ann. Microbiol..

[74] Rohman A., Dijkstra B.W. (2019). The role and mechanism of microbial 3-ketosteroid Δ^1^-dehydrogenases in steroid breakdown. J. Steroid Biochem. Mol. Biol..

[75] Rohman A., Van Oosterwijk N., Thunnissen A.M.W.H., Dijkstra B.W. (2013). Crystal structure and site-directed mutagenesis of 3-ketosteroid Δ^1^-dehydrogenase from *Rhodococcus erythropolis* SQ1 explain its catalytic mechanism. J. Biol. Chem..

[76] Itagaki E., Matushita H., Hatta T. (1990). Steroid transhydrogenase activity of 3-ketosteroid-Δ^1^-dehydrogenase from *Nocardia corallina*. J. Biochem..

[77] Jerussif R., Ringold H.J. (1965). The mechanism of the bacterial C-1,2 dehydrogenation of steroids. III. Kinetics and isotope effects. Biochemistry.

[78] Feller F.M., Richtsmeier P., Wege M., Philipp B. (2021). Comparative analysis of bile-salt degradation in *Sphingobium* sp. strain Chol11 and *P. stutzeri* Chol1 reveals functional diversity of *β*-proteobacterial steroid degradation enzymes and suggests a novel reaction sequence for side-chain degradation involving a hydroxylation step. bioRxiv.

[79] Jones B.V., Begley M., Hill C., Gahan C.G.M., Marchesi J.R. (2008). Functional and comparative metagenomic analysis of bile salt hydrolase activity in the human gut microbiome. Proc. Natl. Acad. Sci. USA.

[80] Rösch V., Denger K., Schleheck D., Smits T.H.M., Cook A.M. (2008). Different bacterial strategies to degrade taurocholate. Arch. Microbiol.

[81] Dong Z., Lee B.H. (2018). Bile salt hydrolases: Structure and function, substrate preference, and inhibitor development. Protein Sci..

[82] Casabon I., Crowe A.M., Liu J., Eltis L.D. (2013). FadD3 is an acyl-CoA synthetase that initiates catabolism of cholesterol rings C and D in Actinobacteria. Mol. Microbiol..

[83] Warnke M., Jung T., Jacoby C., Agne M., Feller F.M., Philipp B., Seiche W., Breit B., Boll M. (2018). Functional characterization of three specific acyl-coenzyme A synthetases involved in anaerobic cholesterol degradation in *Sterolibacterium denitrificans* Chol1S. Appl. Environ. Microbiol..

[84] Holert J., Yücel O., Jagmann N., Prestel A., Möller H.M., Philipp B. (2016). Identification of bypass reactions leading to the formation of one central steroid degradation intermediate in metabolism of different bile salts in *Pseudomonas* sp. strain Chol1. Environ. Microbiol..

[85] Wipperman M.F., Yang M., Thomas S.T., Sampson N.S. (2013). Shrinking the *fadE* proteome of *Mycobacterium tuberculosis*: Insights into cholesterol metabolism through identification of an *α*_2_*β*_2_ heterotetrameric acyl coenzyme A dehydrogenase family. J. Bacteriol..

[86] Yang M., Lu R., Guja K.E., Wipperman M.F., St. Clair J.R., Bonds A.C., Garcia-Diaz M., Sampson N.S. (2016). Unraveling cholesterol catabolism in *Mycobacterium tuberculosis*: ChsE4-ChsE5 *α*_2_*β*_2_ acyl-CoA dehydrogenase initiates *β*-oxidation of 3-oxo-cholest-4-en-26-oyl CoA. ACS Infect. Dis..

[87] Stirling A.J., Gilbert S.E., Conner M., Mallette E., Kimber M.S., Seah S.Y.K. (2020). A key glycine in bacterial steroid-degrading acyl-CoA dehydrogenases allows flavin-ring repositioning and modulates substrate side chain specificity. Biochemistry.

[88] Ruprecht A., Maddox J., Stirling A.J., Visaggio N., Seah S.Y.K. (2015). Characterization of novel acyl coenzyme A dehydrogenases involved in bacterial steroid degradation. J. Bacteriol..

[89] Pidugu L.S., Maity K., Ramaswamy K. (2009). Analysis of proteins with the “Hot dog” fold: Prediction of function and identification of catalytic residues of hypothetical proteins. BMC Struct. Biol..

[90] Haußmann U., Wolters D.A., Fränzel B., Eltis L.D., Poetsch A. (2013). Physiological adaptation of the *Rhodococcus jostii* RHA1 membrane proteome to steroids as growth substrates. J. Proteome Res..

[91] Haapalainen A.M., Meriläinen G., Wierenga R.K. (2006). The thiolase superfamily: Condensing enzymes with diverse reaction specificities. Trends Biochem. Sci..

[92] Schaefer C.M., Lu R., Nesbitt N.M., Schiebel J., Sampson N.S., Kisker C. (2015). FadA5 a thiolase from *Mycobacterium tuberculosis*: A steroid-binding pocket reveals the potential for drug development against tuberculosis. Structure.

[93] Lu R., Schaefer C.M., Nesbitt N.M., Kuper J., Kisker C., Sampson N.S. (2017). Catabolism of the cholesterol side chain in *Mycobacterium tuberculosis* is controlled by a redox-sensitive thiol switch. ACS Infect. Dis..

[94] Casabon I., Swain K., Crowe A.M., Eltis L.D., Mohn W.W. (2014). Actinobacterial acyl coenzyme A synthetases involved in steroid side-chain catabolism. J. Bacteriol..

[95] Gilbert S., Hood L., Seah S.Y.K. (2018). Characterization of an aldolase involved in cholesterol side chain degradation in *Mycobacterium tuberculosis*. J. Bacteriol..

[96] Thomas S.T., Sampson N.S. (2013). *Mycobacterium tuberculosis* utilizes a unique heterotetrameric structure for dehydrogenation of the cholesterol side chain. Biochemistry.

[97] Szentirmai A. (1990). Microbial physiology of sidechain degradation of sterols. J. Ind. Microbiol..

[98] Aggett R., Mallette E., Gilbert S.E., Vachon M.A., Schroeter K.L., Kimber M.S., Seah S.Y.K. (2019). The steroid side-chain–cleaving aldolase Ltp2–ChsH2DUF35 is a thiolase superfamily member with a radically repurposed active site. J. Biol. Chem..

[99] Yang M., Guja K.E., Thomas S.T., Garcia-Diaz M., Sampson N.S. (2014). A distinct MaoC-like enoyl-CoA hydratase architecture mediates cholesterol catabolism in *Mycobacterium tuberculosis*. ACS Chem. Biol..

[100] Yuan T., Yang M., Gehring K., Sampson N.S. (2019). *Mycobacterium tuberculosis* exploits a heterohexameric enoyl-CoA hydratase retro-aldolase complex for cholesterol catabolism. Biochemistry.

[101] Park R.J., Dunn N.W., Ide J.A., Park J., Dunn N.W., Ideb J.A., Park R.J., Dunn N.W., Ide J.A. (1986). A catecholic 9,10*-seco* steroid as a product of aerobic catabolism of cholic acid by a *Pseudomonas* sp.. Steroids.

[102] Van Der Geize R., Hessels G.I., Van Gerwen R., Van Der Meijden P., Dijkhuizen L. (2002). Molecular and functional characterization of *kshA* and *kshB*, encoding two components of 3-ketosteroid 9*α*-hydroxylase, a class IA monooxygenase, in *Rhodococcus erythropolis* strain SQ1. Mol. Microbiol..

[103] Petrusma M., Dijkhuizen L., Van Der Geize R. (2009). *Rhodococcus rhodochrous* DSM 43269 3-ketosteroid 9*α*-hydroxylase, a two-component iron-sulfur-containing monooxygenase with subtle steroid substrate specificity. Appl. Environ. Microbiol..

[104] Petrusma M., Hessels G., Dijkhuizen L., van der Geize R. (2011). Multiplicity of 3-ketosteroid-9*α*-hydroxylase enzymes in *Rhodococcus rhodochrous* DSM43269 for specific degradation of different classes of steroids. J. Bacteriol..

[105] Penfield J.S., Worrall L.J., Strynadka N.C., Eltis L.D. (2014). Substrate specificities and conformational flexibility of 3-ketosteroid 9*α*-hydroxylases. J. Biol. Chem..

[106] Ibero J., Galán B., Díaz E., García J.L. (2019). Testosterone degradative pathway of *Novosphingobium tardaugens*. Genes.

[107] Dagley S. (1971). Catabolism of aromatic compounds by micro-organisms. Adv. Microbial. Physiol..

[108] Horinouchi M., Hayashi T., Kudo T. (2004). The genes encoding the hydroxylase of 3-hydroxy-9,10-*seco*androsta-1,3,5(10) -triene-9,17-dione in steroid degradation in *Comamonas testosteroni* TA441. J. Steroid Biochem. Mol. Biol..

[109] Horinouchi M., Yamamoto T., Taguchi K., Arai H., Kudo T. (2001). *Meta*-cleavage enzyme gene *tesB* is necessary for testosterone degradation in *Comamonas testosteroni* TA441. Microbiology.

[110] Yam K.C., D’Angelo I., Kalscheuer R., Zhu H., Wang J.X., Snieckus V., Ly L.H., Converse P.J., Jacobs W.R., Strynadka N. (2009). Studies of a ring-cleaving dioxygenase illuminate the role of cholesterol metabolism in the pathogenesis of *Mycobacterium tuberculosis*. PLoS Pathog..

[111] Van der Geize R., Yam K., Heuser T., Wilbrink M.H., Hara H., Anderton M.C., Sim E., Dijkhuizen L., Davies J.E., Mohn W.W. (2007). A gene cluster encoding cholesterol catabolism in a soil Actinomycete provides insight into *Mycobacterium tuberculosis* survival in macrophages. Proc. Natl. Acad. Sci. USA.

[112] Horinouchi M., Hayashi T., Koshino H., Yamamoto T., Kudo T. (2003). Gene encoding the hydrolase for the product of the *meta*-cleavage reaction in testosterone degradation by *Comamonas testosteroni*. Appl. Environ. Microbiol..

[113] Seah S.Y.K., Ke J., Denis G., Horsman G.P., Fortin P.D., Whiting C.J., Eltis L.D. (2007). Characterization of a C-C bond hydrolase from *Sphingomonas wittichii* RW1 with novel specificities towards polychlorinated biphenyl metabolites. J. Bacteriol..

[114] Lack N., Lowe E.D., Liu J., Eltis L.D., Noble M.E.M., Sim E., Westwood I.M. (2007). Structure of HsaD, a steroid-degrading hydrolase, from *Mycobacterium tuberculosis*. Acta Crystallogr. Sect. F Struct. Biol. Cryst. Commun..

[115] Lack N.A., Yam K.C., Lowe E.E., Horsman G.P., Owen R.L., Sim E., Eltis L.D. (2010). Characterization of a carbon-carbon hydrolase from M*ycobacterium tuberculosis* involved in cholesterol metabolism. J. Biol. Chem..

[116] Horinouchi M., Hayashi T., Koshino H., Kurita T., Kudo T. (2005). Identification of 9,17-dioxo-1,2,3,4,10,19-hexanorandrostan-5-oic acid, 4-hydroxy-2-oxohexanoic acid, and 2-hydroxyhexa-2,4-dienoic acid and related enzymes involved in testosterone degradation in *Comamonas testosteroni* TA441. Appl. Environ. Microbiol..

[117] Carere J., McKenna S.E., Kimber M.S., Seah S.Y.K. (2013). Characterization of an aldolase-dehydrogenase complex from the cholesterol degradation pathway of *Mycobacterium tuberculosis*. Biochemistry.

[118] Fahrbach M., Krauss M., Preiss A., Kohler H.P.E., Hollender J. (2010). Anaerobic testosterone degradation in *Steroidobacter denitrificans*-identification of transformation products. Environ. Pollut..

[119] Fahrbach M., Kuever J., Remesch M., Huber B.E., Kämpfer P., Dott W., Hollender J. (2008). *Steroidobacter denitrificans* gen. nov., sp. nov., a steroidal hormone-degrading *γ*-Proteobacterium. Int. J. Syst. Evol. Microbiol..

[120] Tarlera S., Denner E.B.M. (2003). *Sterolibacterium denitrificans* gen. nov., sp. nov., a novel cholesterol-oxidizing, denitrifying member of the *β*-Proteobacteria. Int. J. Syst. Evol. Microbiol..

[121] Shih C.-J., Chen Y.-L., Wang C.-H., Wei S.T.-S., Lin I.-T., Ismail W.A., Chiang Y.-R. (2017). Biochemical mechanisms and microorganisms involved in anaerobic testosterone metabolism in estuarine sediments. Front. Microbiol..

[122] Wang P.H., Yu C.P., Lee T.H., Lin C.W., Ismail W., Wey S.P., Kuo A.T., Chiang Y.R. (2014). Anoxic androgen degradation by the denitrifying bacterium *Sterolibacterium denitrificans* via the 2,3-*seco* pathway. Appl. Environ. Microbiol..

[123] Wang P.-H., Leu Y.-L., Ismail W., Tang S.-L., Tsai C.-Y., Chen H.-J., Kao A.-T., Chiang Y.-R. (2013). Anaerobic and aerobic cleavage of the steroid core ring structure by *Steroidobacter denitrificans*. J. Lipid Res..

[124] Yang F.-C., Chen Y.-L., Tang S.-L., Yu C.-P., Wang P.-H., Ismail W., Wang C.-H., Ding J.-Y., Yang C.-Y.C.-Y., Yang C.-Y.C.-Y. (2016). Integrated multi-omics analyses reveal the biochemical mechanisms and phylogenetic relevance of anaerobic androgen biodegradation in the environment. ISME J..

[125] Crowe A.M., Casabon I.I., Brown K.L., Liu J., Lian J., Rogalski J.C., Hurst T.E., Snieckus V., Foster L.J., Eltis L.D. (2017). Catabolism of the last two steroid rings in *Mycobacterium tuberculosis* and other bacteria. mBio.

[126] Gadbery J., Round J., Yuan T., Wipperman M.F., Story K.T., Crowe A., Casabon I., Liu J., Yang X., Eltis L.D. (2020). IpdE1-IpdE2 is a heterotetrameric acyl coenzyme A dehydrogenase that is widely distributed in steroid-degrading bacteria. Biochemistry.

[127] Horinouchi M., Hayashi T., Koshino H., Malon M., Hirota H., Kudo T. (2014). Identification of 9*α*-hydroxy-17-oxo-1,2,3,4,10,19-hexanorandrostan- 5-oic acid in steroid degradation by *Comamonas testosteroni* TA441 and its conversion to the corresponding 6-en-5-oyl coenzyme A (CoA) involving open reading frame 28 (ORF28)- and ORF30-encoded acyl-CoA dehydrogenases. J. Bacteriol..

[128] Horinouchi M., Koshino H., Malon M., Hirota H., Hayashi T. (2018). Steroid degradation in *Comamonas testosteroni* TA441: Identification of metabolites and the genes involved in the reactions necessary before D-ring cleavage. Appl. Environ. Microbiol..

[129] Horinouchi M., Malon M., Hirota H., Hayashi T. (2019). Identification of 4-methyl-5-oxo-octane-1,8-dioic acid and the derivatives as metabolites of steroidal **C,D**-ring degradation in *Comamonas testosteroni* TA441. J. Steroid Biochem. Mol. Biol..

[130] Horinouchi M., Koshino H., Malon M., Hirota H., Hayashi T. (2019). Identification of 9-oxo-1,2,3,4,5,6,10,19-octanor-13,17*-seco*androst-8(14)-ene-7,17-dioic acid as a metabolite of steroid degradation in *Comamonas testosteroni* TA441 and the genes involved in the conversion. J. Steroid Biochem. Mol. Biol..

[131] Helenius A., Simons K. (1975). Solubilization of membranes by detergents. BBA Rev. Biomembr..

[132] Begley M., Gahan C.G.M., Hill C. (2005). The interaction between bacteria and bile. FEMS Microbiol. Rev..

[133] Bernstein C., Bernstein H., Payne C.M., Beard S.E., Schneider J. (1999). Bile salt activation of stress response promoters in *Escherichia coli*. Curr. Microbiol..

[134] Cremers C.M., Knoefler D., Vitvitsky V., Banerjee R., Jakob U. (2014). Bile salts act as effective protein-unfolding agents and instigators of disulfide stress in vivo. Proc. Natl. Acad. Sci. USA.

[135] Gunn J.S. (2000). Mechanisms of bacterial resistance and response to bile. Microbes Infect..

[136] Nikaido H. (2003). Molecular basis of bacterial outer membrane permeability revisited. Microbiol. Mol. Biol. Rev..

[137] Hancock R.E.W. (1997). The bacterial outer membrane as a drug barrier. Trends Microbiol..

[138] Paul S., Alegre K.O., Holdsworth S.R., Rice M., Brown J.A., Mcveigh P., Kelly S.M., Law C.J. (2014). A single-component multidrug transporter of the major facilitator superfamily is part of a network that protects *Escherichia coli* from bile salt stress. Mol. Microbiol..

[139] Lee C.Y., Liu W.H. (1992). Production of androsta-1,4-diene-3,17-dione from cholesterol using immobilized growing cells of *Mycobacterium* sp. NRRL B-3683 adsorbed on solid carriers. Appl. Microbiol. Biotechnol..

[140] Perez C., Falero A., Llanes N., Hung B.R., Hervé M.E., Palmero A., Martí E. (2003). Resistance to androstanes as an approach for androstandienedione yield enhancement in industrial mycobacteria. J. Ind. Microbiol. Biotechnol..

[141] Göhler A., Xiong G., Paulsen S., Trentmann G., Maser E. (2008). Testosterone-inducible regulator is a kinase that drives steroid sensing and metabolism in *Comamonas testosteroni*. J. Biol. Chem..

[142] Gong W., Xiong G., Maser E. (2012). Oligomerization and negative autoregulation of the LysR-type transcriptional regulator HsdR from *Comamonas testosteroni*. J. Steroid Biochem. Mol. Biol..

[143] Kendall S.L., Burgess P., Balhana R., Withers M., Ten Bokum A., Lott J.S., Gao C., Uhia-Castro I., Stoker N.G. (2010). Cholesterol utilization in mycobacteria is controlled by two TetR-type transcriptional regulators: *kstR* and *kstR2*. Microbiology.

[144] García-Fernández E., Medrano F.J., Galán B., García J.L. (2014). Deciphering the transcriptional regulation of cholesterol catabolic pathway in mycobacteria: Identification of the inducer of KstR repressor. J. Biol. Chem..

[145] Casabon I., Zhu S.H., Otani H., Liu J., Mohn W.W., Eltis L.D. (2013). Regulation of the KstR2 regulon of *Mycobacterium tuberculosis* by a cholesterol catabolite. Mol. Microbiol..

[146] Taoka H., Yokoyama Y., Morimoto K., Kitamura N., Tanigaki T., Takashina Y., Tsubota K., Watanabe M. (2016). Role of bile acids in the regulation of the metabolic pathways. World J. Diabetes July World J. Diabetes.

[147] Chiang J.Y.L. (2013). Bile acid metabolism and signaling. Compr. Physiol..

[148] Li W. (2002). Bile acid secreted by male sea lamprey that acts as a sex pheromone. Science.

[149] Li K., Scott A.M., Brant C.O., Fissette S.D., Riedy J.J., Hoye T.R., Li W. (2017). Bile salt-like dienones having a novel skeleton or a rare substitution pattern function as chemical cues in adult sea lamprey. Org. Lett..

[150] Brant C.O., Chung-Davidson Y.-W., Li K., Scott A.M., Li W. (2013). Biosynthesis and release of pheromonal bile salts in mature male sea lamprey. BMC Biochem..

[151] Zhang Z., Zhang Q., Dexheimer T.S., Ren J., Neubig R.R., Li W. (2020). Two highly related odorant receptors specifically detect a-bile acid pheromones in sea lamprey (*Petromyzon marinus*). J. Biol. Chem..

[152] Hahn M.A., Effertz C., Bigler L., von Elert E. (2019). 5*α*-cyprinol sulfate, a bile salt from fish, induces diel vertical migration in *Daphnia*. Elife.

[153] Doyle W.I., Dinser J.A., Cansler H.L., Zhang X., Dinh D.D., Browder N.S., Riddington I.M., Meeks J.P. (2016). Faecal bile acids are natural ligands of the mouse accessory olfactory system. Nat. Commun..

[154] Li K., Brant C.O., Siefkes M.J., Kruckman H.G., Li W. (2013). Characterization of a novel bile alcohol sulfate released by sexually mature male sea lamprey (*Petromyzon marinus*). PLoS ONE.

[155] Schulenburg H., Félix M.A. (2017). The natural biotic environment of *Caenorhabditis elegans*. Genetics.

[156] Frézal L., Félix M.A. (2015). *C. elegans* outside the Petri dish. Elife.

[157] Maglich J.M., Sluder A., Guan X., Shi Y., McKee D.D., Carrick K., Kamdar K., Willson T.M., Moore J.T. (2001). Comparison of complete nuclear receptor sets from the human, *Caenorhabditis elegans* and *Drosophila* genomes. Genome Biol..

[158] Motola D.L., Cummins C.L., Rottiers V., Sharma K.K., Li T., Li Y., Suino-Powell K., Xu H.E., Auchus R.J., Antebi A. (2006). Identification of ligands for DAF-12 that govern dauer formation and reproduction in *C. elegans*. Cell.

[159] Aguilaniu H., Fabrizio P., Witting M. (2016). The role of dafachronic acid signaling in development and longevity in *Caenorhabditis elegans*: Digging deeper using cutting-edge analytical chemistry. Front. Endocrinol. Lausanne.

[160] Mansell D.S., Bryson R.J., Harter T., Webster J.P., Kolodziej E.P., Sedlak D.L. (2011). Fate of endogenous steroid hormones in steer feedlots under simulated rainfall-induced runoff. Environ. Sci. Technol.

[161] Gravert T.K.O., Fauser P., Olsen P., Hansen M. (2021). In situ formation of environmental endocrine disruptors from phytosterol degradation: A temporal model for agricultural soils. Environ. Sci. Process. Impacts.

[162] Matsumoto A.M., Marck B.T. (2006). DEA Agreement No. DEA-04- P0007 Final Report [Analysis of the Androgenic and Anabolic Activities of 1,4-Androstadien-3,17-dione and 19-nor-4,9(10)- Androstadienedione in Male Sprague Dawley Rats]. DEA Document ID DEA-2008-0007-0003. https://www.regulations.gov/document/DEA-2008-0007-0003.

[163] Bhasin S., Jasuja R. (2005). Pharmacological Analysis of Boldione and 19-nor-4,9(10)-Androstadienedione for Androgenic Activity Using C3H10T1/2 Stem Cells. DEA Document ID DEA-2008-0007-0002. https://www.regulations.gov/document/DEA-2008-0007-0002.

[164] Barceloux D.G., Palmer R.B. (2013). Anabolic—Androgenic steroids. Disease-a-Month.

[165] Kicman A. (2008). Pharmacology of anabolic steroids. Br. J. Pharmacol..

[166] Hou L., Xie Y., Ying G., Fang Z. (2011). Developmental and reproductive characteristics of western mosquitofish (*Gambusia affinis*) exposed to paper mill effluent in the Dengcun River, Sihui, South China. Aquat. Toxicol..

[167] Brockmeier E.K., Jayasinghe B.S., Pine W.E., Wilkinson K.A., Denslow N.D. (2014). Exposure to paper mill effluent at a site in north central Florida elicits molecular-level changes in gene expression indicative of progesterone and androgen exposure. PLoS ONE.

[168] Parks L.G., Lambright C.S., Orlando E.F., Guillette L.J., Ankley G.T., Gray L.E. (2001). Masculinization of female mosquitofish in kraft mill effluent-contaminated fenholloway river water is associated with androgen receptor agonist activity. Toxicol. Sci..

[169] Carson J., Jenkins R., Wilson E., Howell W., Moore R. (2007). Naturally occurring progesterone in Loblolly pine: A major steroid precursor of environmental androgens. Environ. Toxicol. Chem..

[170] Dlugovitzky D.G., Fontela M.S., Martinel Lamas D.J., Valdez R.A., Romano M.C. (2015). *Mycobacterium smegmatis* synthesizes in vitro androgens and estrogens from different steroid precursors. Can. J. Microbiol..

[171] Jenkins R.L., Wilson E.M., Angus R.A., Howell W.M., Kirk M., Moore R., Nance M., Brown A. (2004). Production of androgens by microbial transformation of progesterone in vitro: A model for androgen production in rivers receiving paper mill effluent. Environ. Health Perspect..

[172] Enmark E., Gustafsson J.Å. (2000). Nematode genome sequence dramatically extends the nuclear receptor superfamily. Trends Pharmacol. Sci..

[173] Sluder A.E., Mathews S.W., Hough D., Yin V.P., Maina C.V. (1999). The nuclear receptor superfamily has undergone extensive proliferation and diversification in nematodes. Genome Res..

[174] Taubert S., Ward J.D., Yamamoto K.R. (2011). Nuclear hormone receptors in nematodes: Evolution and function. Mol. Cell. Endocrinol..

[175] Donova M.V., Egorova O.V. (2012). Microbial steroid transformations: Current state and prospects. Appl. Microbiol. Biotechnol..

[176] Bortolini O., Medici A., Poli S. (1997). Biotransformations on steroid nucleus of bile acids. Steroids.

[177] Bouchier I.A.D. (1980). The medical treatment of gallstones. Ann. Rev. Med..

[178] Beuers U., Spengler U., Kruis W., Aydemir Ü., Wiebecke B., Heldwein W., Weinzierl M., Pape G.R., Sauerbruch T., Paumgartner G. (1992). Ursodeoxycholic acid for treatment of primary sclerosing cholangitis: A placebo-controlled trial. Hepatology.

[179] Kim E., Cho J.H., Kim E., Kim Y.J. (2017). Ursodeoxycholic acid inhibits the proliferation of colon cancer cells by regulating oxidative stress and cancer stem-like cell growth. PLoS ONE.

[180] Braun M., Sun B., Anselment B., Weuster-Botz D. (2012). Novel whole-cell biocatalysts with recombinant hydroxysteroid dehydrogenases for the asymmetric reduction of dehydrocholic acid. Appl. Microbiol. Biotechnol..

[181] Bordoni A., Amaretti A., Leonardi A., Boschetti E., Danesi F., Matteuzzi D., Roncaglia L., Raimondi S., Rossi M. (2013). Cholesterol-lowering probiotics: In vitro selection and in vivo testing of bifidobacteria. Appl. Microbiol. Biotechnol..

[182] Ooi L.G., Liong M.T. (2010). Cholesterol-lowering effects of probiotics and prebiotics: A review of in vivo and in vitro findings. Int. J. Mol. Sci..

[183] Kumar R., Grover S., Batish V.K. (2011). Hypocholesterolaemic effect of dietary inclusion of two putative probiotic bile salt hydrolase-producing *Lactobacillus plantarum* strains in Sprague–Dawley rats. Br. J. Nutr..

